# A Subjective and Intuitive Approach to Rapid, Holistic Assessment of Natural Ecosystem Integrity Across a Community‐Managed Conservation Area in Southern Tanzania

**DOI:** 10.1002/ece3.70872

**Published:** 2025-03-02

**Authors:** Lily M. Duggan, Katrina A. Walsh, Lucia J. Tarimo, Deogratius R. Kavishe, Ramiro D. Crego, Manase Elisa, Fidelma Butler, Felister Mombo, Gerry F. Killeen

**Affiliations:** ^1^ School of Biological Earth & Environmental Sciences University College Cork Cork Republic of Ireland; ^2^ Environmental Research Institute University College Cork, Cork, Environmental Research Institute Cork Republic of Ireland; ^3^ Department of Environmental Health and Ecological Sciences Ifakara Health Institute Morogoro Tanzania; ^4^ College of Forestry, Wildlife and Tourism Sokoine University of Agriculture Morogoro Tanzania; ^5^ Tanzania National Parks Arusha Tanzania

**Keywords:** biodiversity, encroachment, habitat, mammal, management, species richness

## Abstract

Quantitative surveys of wild animal abundance or activity, and assessments of the integrity of the complex natural ecosystems they live in, are typically quite laborious and meaningful analysis of the data obtained may require considerable time and expertise. This study describes the development and evaluation of a practical procedure for semi‐quantitative consensus‐based synthesis of subjective impressions accumulated by a small team of investigators who visited 32 different locations distributed in or around a community‐based Wildlife Management Area in southern Tanzania. The *subjective natural ecosystem integrity index* (SNEII) scores obtained represent a holistic indicator of all aspects of land use, wildlife and human activities, which correlated strongly with objective indicators of wild animal community or whole natural ecosystem integrity that were estimated directly from quantitative survey data by the same investigators at the same locations. Also, comparative regression analysis indicated that the SNEII was a far more sensitive to variations in observed human activities than any of the objective alternatives, correspondingly yielding far more detailed insights into ongoing conservation challenges. This simple procedure for summarizing the overall, multi‐faceted subjective impressions of individuals traversing extensive conservation areas may well be applicable through participatory approaches to routine programmatic monitoring by community‐based staff with minimal training, and may therefore be more practically useful to devolved conservation areas like WMAs than conventional objective statistical synthetic indices relying on laborious collection and expert analysis of quantitative survey data.

## Graphical abstract

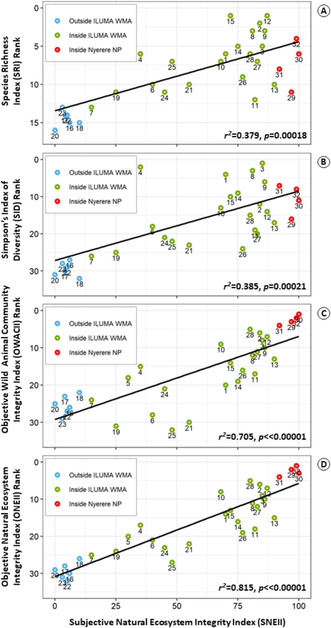

## 1 Introduction

The aim of community‐based conservation initiatives over recent decades has been to shift from exclusively state‐controlled “fortress conservation” (Brockington 2002) to devolved, inclusive, bottom‐up, community‐led management (Goldman 2003). However, since the outset of this broad effort (Gibson and Marks 1995; Agrawal and Ribot 1999), many such community‐based conservation initiatives, including the Wildlife Management Areas (WMAs) of Tanzania (Mwakaje et al. 2013; Noe and Kangalawe 2015; Bluwstein, Moyo, and Kicheleri 2016; Wright 2017; Kicheleri et al. 2021; Kicheleri et al. 2018; Keane et al. 2020, Raycraft 2022), have struggled to contend with competing land use pressures, establishment of effective, genuinely bottom‐up democratic governance functions and attracting sufficient income, especially since the dramatic contraction of trophy‐hunting tourism in recent years (Dickman et al. 2019).

Another key obstacle to be overcome has been the paucity of practical monitoring procedures that are practically accessible, affordable and meaningful for the communities who take on such devolved stewardship roles, while also being sufficiently robust and standardizable to be useful to higher‐level national stakeholders and external actors (Bennun et al. 2005; Danielsen, Burgess, and Balmford 2005; Setty et al. 2008; Vergara‐Asenjo, Sharma, and Potvin 2015; Evans, Guariguata, and Brancalion 2018; Kusters et al. 2018; Pocock et al. 2019; Mandeville, Nilsen, and Finstad 2022; Beaudoin et al. 2016; Mandeville et al. 2023). Unfortunately, formally structured quantitative surveys of wild animal abundance, activity or biodiversity, or indeed broader assessments of the functionality of the complex natural ecosystems they live in, are often laborious and expensive to apply in low‐income contexts (Danielsen, Burgess, and Balmford 2005; Poulsen and Luanglath 2005; Evans, Guariguata, and Brancalion 2018; Kusters et al. 2018; Mandeville et al. 2023). Furthermore, meaningful statistical analysis of the data obtained requires considerable time and expertise, which often lies beyond the technical capabilities and budgets that such small‐to‐medium scale community‐based programmes can sustain outside of time‐limited projects (Danielsen, Burgess, and Balmford 2005; Poulsen and Luanglath 2005; Setty et al. 2008; Mandeville et al. 2023). Also, members of local communities often consider such survey methods rather abstract, impractical, one‐dimensional and forced upon them from outside, lacking satisfactory nuance or responsiveness to local needs (Bennun et al. 2005; Danielsen, Burgess, and Balmford 2005; Setty et al. 2008; Vergara‐Asenjo, Sharma, and Potvin 2015; Evans, Guariguata, and Brancalion 2018; Kusters et al. 2018; Beaudoin et al. 2016; Mandeville et al. 2023). Indeed, stakeholder communities typically take a much more holistic view of conservation, natural resource use and rural development priorities that needs to be integrated into devolved conservation schemes in a multifaceted and bottom‐up manner that starts with them at the grass roots level management (Bennun et al. 2005; Danielsen, Burgess, and Balmford 2005; Setty et al. 2008; Vergara‐Asenjo, Sharma, and Potvin 2015; Evans, Guariguata, and Brancalion 2018; Kusters et al. 2018; Pocock et al. 2019; Beaudoin et al. 2016; Mandeville et al. 2023). Furthermore, incorporating such subjective, often qualitative in‐depth community knowledge can yield invaluable scientific insights that are otherwise missed by more conventional, supposedly objective, quantitative research methodologies (Raddaoui 2009; Vergara‐Asenjo, Sharma, and Potvin 2015; Beaudoin et al. 2016).

As a result, considerable research investment over the last two decades has focused on the development of participatory approaches to mapping, monitoring and evaluation for conservation programmes that not only tap into the full depth and breadth of community knowledge but also engage them more effectively as the primary stewards and beneficiaries of conserved areas management (Bennun et al. 2005; Setty et al. 2008; Evans, Guariguata, and Brancalion 2018; Kusters et al. 2018; Pocock et al. 2019; Thiao et al. 2019; Mandeville et al. 2023). Learning from past mistakes with top‐down approaches that sidelined communities (Agrawal and Ribot 1999; Mwakaje et al. 2013; Bluwstein, Moyo, and Kicheleri 2016; Wright 2017; Kicheleri et al. 2021; Kicheleri et al. 2018; Keane et al. 2020; Raycraft 2022) such efforts to develop *multi‐stakeholder platforms* for *integrated landscape initiatives* require mapping and monitoring procedures that are both meaningful and practically useful to grass roots local stakeholders for routine management (Kusters et al. 2018). Such participatory methodologies also need to be broadly applicable and standardizable enough to enable reliable comparative evaluations of multiple independently run local schemes distributed across national, and ideally international geographic scales, as well as time trends extending over decades rather than just years (Setty et al. 2008; Kusters et al. 2018).

This study describes the development and evaluation of a practical procedure for numerical synthesis of consensus subjective impressions accumulated by three professional researchers, escorted by a team of locally recruited Village Game Scouts, in and around a community‐based Wildlife Management Area in southern Tanzania (Duggan 2023), to yield a semi‐quantitative index of natural ecosystem integrity. This perception‐based indicator of all aspects of land use, wildlife and human activities, was then evaluated through statistical comparisons with four different conventional indicators of wild animal community or whole natural ecosystem integrity that were estimated directly from quantitative survey data at the same times and places (Ethical approval permit numbers: Tanzania Wildlife Management Authority: No. TWRI/RS/364/11, COSTECH: 2022‐191‐NA‐2022‐053, Institutional Review Board of the Ifakara Health Institute: IHI/IRB/5–2021, Medical Research Coordination Committee of the National Institute for Medical Research: NIMR/HQ/R.8a/Vol.IX/3719, Animal Experimentation Ethics Committee of University College Cork: 21–001).

## 2 Materials and Methods

### 2.1 Study Area Geography and Conservation Management Model

**FIGURE 1:**
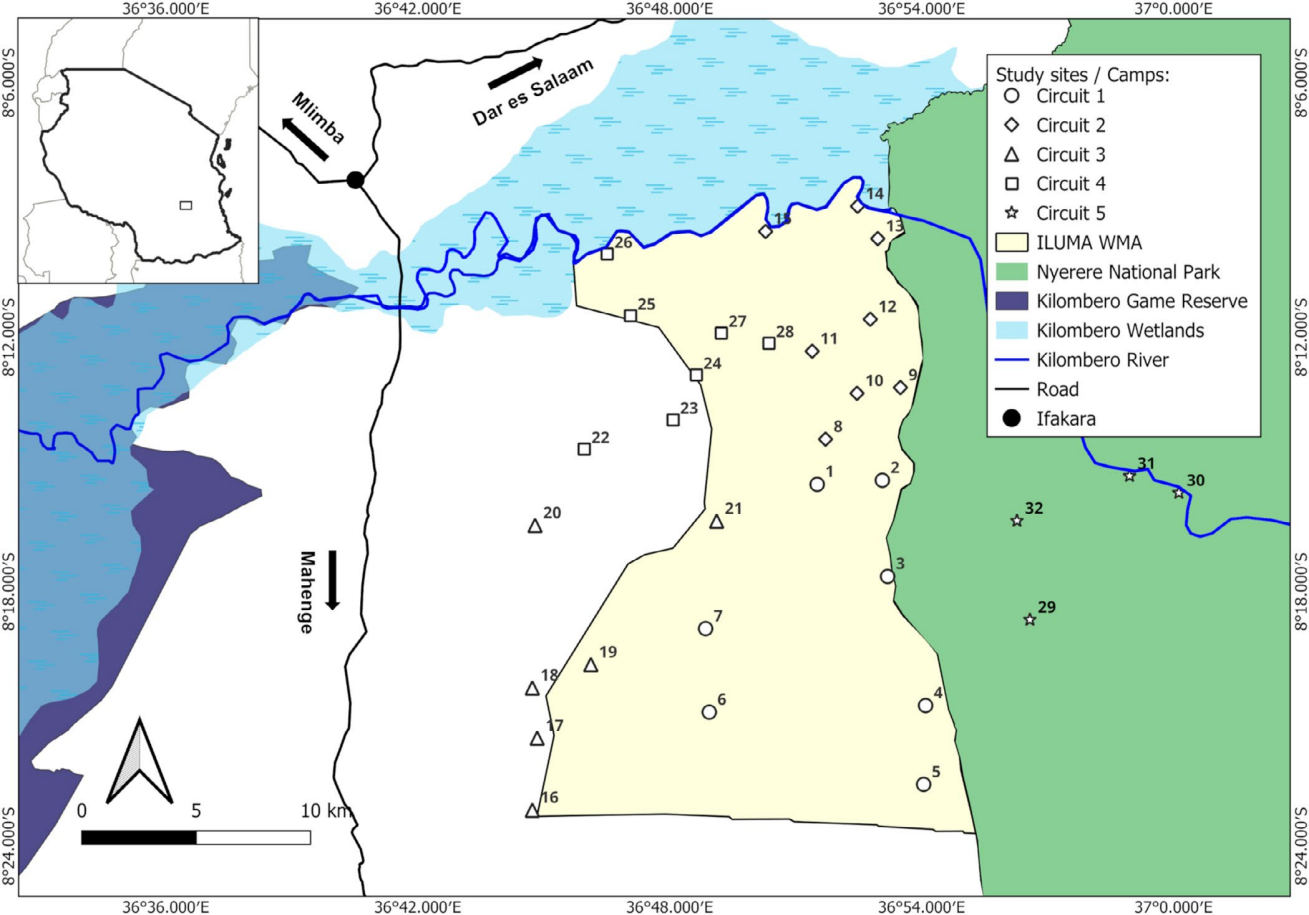
Map of the study area in its local and national context in southern Tanzania. All of the 32 camps detailed in Data S1 are illustrated in the geographic context of the Ifakara‐Lupiro‐Mangula Wildlife Management Area (ILUMA WMA), Nyerere National Park (NNP), Udzungwa Mountains National Park, Ifakara town and the extensive seasonal wetlands of the Kilombero river (Daconto et al. 2018). The climate of the region is moist and tropical with distinct seasons: The season of reasonably consistent heavy rainfall occurs between March and May each year, with the dry season from June through to October generally experiencing negligible rainfall, and a short season of more sporadic rains occurring between November and February. Annual total rainfall in the Kilombero valley typically averages between 1200 and 14,000 mm (Mombo et al. 2011) and temperatures are highest from December to March.

This study was conducted in and around the Ifakara‐Lupiro‐Mangula (ILUMA) WMA in southern Tanzania (Figure 1). WMAs are intended as a model mechanism for community‐based conservation in Tanzania, which should enable devolution of authority for the management of protected community lands to local stakeholder communities, while also enabling them to leverage sustainable livelihoods and collectively managed income for local rural development initiatives (USAID 2018). Each WMA has its own individual set of by‐laws to permit and regulate sustainable utilization practices for selected natural resources, with fishing on the Kilombero River from human settlements established specifically for this purpose (District Authorities 2016) representing a clearly successful example within ILUMA from which valuable lessons can be learned (Duggan 2023).

Having said that, the overwhelming majority of Tanzania WMAs have failed to achieve financial sustainability thus far, resulting in little if any benefit to local communities (Mwakaje et al. 2013; Noe and Kangalawe 2015; Bluwstein, Moyo, and Kicheleri 2016; Wright 2017; Keane et al. 2020; Raycraft 2022). Furthermore, even the most successful examples have well‐documented governance limitations in terms of lack of *de facto* local ownership and control (Mwakaje et al. 2013; Bluwstein, Moyo, and Kicheleri 2016; Wright 2017; Kicheleri et al. 2021; Kicheleri et al. 2018; Keane et al. 2020; Raycraft 2022). Specifically, Village Councils have been systematically sidelined from the their natural roles in oversight and management of WMAs, resulting in local people's access to natural resources being curtailed, while most of the financial benefits have been accrued by the central government and private sector tourism operators (Noe and Kangalawe 2015; Bluwstein, Moyo, and Kicheleri 2016; Wright 2017; Kicheleri et al. 2021; Keane et al. 2020; Raycraft 2022).

Beyond needing to secure and manage sufficient income to the benefit of local communities, WMAs also need to track and manage natural resource use patterns as practically and affordably as possible, in terms that local communities can relate to. Day‐to‐day monitoring and regulation of tourism, forestry, pastoralism and fishing activities within WMAs is therefore carried out by locally resident Village Game Scouts (VGS), who are appointed and paid by the WMA management team as its community‐based equivalent of wildlife rangers (District Authorities 2016). VGS are also responsible for protecting the natural resources of the area against unregulated, unsustainable exploitation, while also protecting people's lives, property and livelihoods against wildlife. WMAs therefore represent ideal settings for piloting new survey methods for programmatic mapping and monitoring applications, because these VGS are well‐placed to support the development of these procedures, incorporate them into their routine activities and adapt them to participatory formats suitable for use by local community members. Furthermore, the legally stipulated governance and management processes of Tanzanian WMAs are broadly representative of those required by most community‐based conservation schemes across most of Africa, so experiences of pilot assessments like this may be useful in a variety of contexts.

The ILUMA WMA includes land from 14 villages from both the Ulanga and Kilombero districts, with stakeholder communities that include more than 80,000 residents (United Republic of Tanzania Ministry of Natural Resources and Tourism 2015). The longest established surviving ethnic groups in the area are the *Pogoro*, traditionally farmers and hunters of the uplands that flank the Kilombero Valley wetlands, and the *Ndamba* who traditionally fished within it. Pastoralists from northern and central Tanzania, largely *Masaii* and *Sukuma*, only began to arrive toward the end of the 20th century (Brehony, Nan'goro, and Sakafu 2001; Moshiro 2010; Daconto et al. 2018).

This steady recent influx of these relatively new communities, who now comprise approximately 30% of the population of the valley (Daconto et al. 2018), has resulted in considerable conflict over land and natural resources that merits careful attention in its own right (Brehony, Nan'goro, and Sakafu 2001; Moshiro 2010; Daconto et al. 2018) and will be squarely addressed in subsequent reports of other complementary studies carried out under the same project. Such complex and sensitive societal challenges need to be carefully explored in a more nuanced, in‐depth manner than can be responsibly accommodated within this report, so its scope is deliberately restricted to the development and assessment of a new procedures for assessing conservation effectiveness in simple terms of the relative integrity of the historical natural ecosystem at any given specific location.

The community lands set aside for collective conservation and management by ILUMA WMA spans a total area of 509km^2^ (United Republic of Tanzania Ministry for Natural Resources and Tourism 2023) at the downstream end of the Kilombero Valley, encompassing both Kilombero and Ulanga districts in the Morogoro region of Southern Tanzania (Figure 1). Covering an area of over 7000km^2^ the inland delta of the Kilombero Valley, most of which lies upstream and to the east of the ILUMA WMA, is the largest lowland freshwater wetland in East Africa and a recognized RAMSAR site (United Republic of Tanzania Ministry for Natural Resources and Tourism 2023). Because of its generally fertile soils and seasonal inundation, the predominant livelihood‐related land use across the valley is rice farming, although sugar cane and other tillage crops, forest products, livestock rearing and fishing also contribute to household incomes (Daconto et al. 2018).

Immediately to the east of ILUMA WMA, Nyerere National Park (NNP) is Africa's largest national park, gazetted quite recently in 2019 and spanning 30,893 km^2^ (Nyerere National Park 2023). While the northern bank of the Kilombero river is included in the protected area of the WMA, otherwise all the areas to the south, west and north contain expanses of agricultural land and floodplains used for mobile livestock herding. ILUMA acts as a buffer zone for NNP and includes zones designated for tourist hunting and local hunting, in addition to a wetland conservation zone where regulated fishing activities are permitted. The natural historical land cover of the community lands designated for conservation by the ILUMA authority to the south of the Kilombero River (Daconto et al. 2018), consists of miombo woodlands, a dense groundwater forest along the southern bank of the Kilombero river, thinner ribbons of riparian forest along various seasonal drainage lines and smaller glades that are inundated during the rainy season, as well as open grasslands in extensive seasonal wetlands dominated by papyrus.

### 2.2 Study Design and Sampling Frame

As explained in detail elsewhere (Duggan et al. 2024; Duggan 2023) this study was carried out using a repeated rolling cross‐sectional design with three rounds of surveys encompassing a total of 32 defined locations, referred to herein as *camps*, that were distributed across NNP, ILUMA WMA and the villages immediately to the west of it (Figure 1 and Data S1), over the course of 1 year. Data were collected in three distinct rounds. Round 1: from 21st January to 16th March 2022. Round 2: from 22nd March to 25th May 2022. Round 3: from 25th August to 29th November 2022. Rounds one and two occurred during the wet season and round 3 during the dry season. Not every camp was visited in each round due to circumstances such as physical inaccessibility during the rains or lack of accessible surface or groundwater for cooking and drinking at the end of the dry season. Round 1 surveyed 20 camps: 17 inside ILUMA and 3 outside. Round 2 surveyed 28 camps: all 22 camps inside ILUMA and 6 camps outside. Round 3 surveyed 28 camps: 18 camps inside ILUMA, 6 outside and 4 in Nyerere National Park.

As explained in detail elsewhere (Duggan et al. 2024; Duggan 2023), the broad geographic distribution of the camps (Figure 1) was planned to encompass as wide of a range of ecosystem states as possible, by including all parts of ILUMA WMA, the neighboring domesticated land to the west and some of parts of NNP immediately to the east (Data S1 and S2). However, the exact position of a camp location for inclusion in the survey sample frame was ultimately determined and recorded in situ, based the requirement for perennial surface water, to not only attract various wildlife, humans and livestock, but also sustain the *Anopheles* mosquito populations relevant to the malaria vector biology objectives of the overall project (Kavishe et al. 2025; Walsh 2023). Each of these camps was assigned to one of five circuits, the first four of which started and ended centrally at *Msakamba* (Camp 1) and roughly corresponded to the southeast, southwest, northeast, and northwest quarters of the ILUMA WMA (Figure 1, Data S1). While the first two rounds of surveys only visited these first four circuits, with a combined total of 28 camp locations, it became obvious at that point that no camp in the WMA lacked signs of human disturbance. A new fifth circuit comprising four new, essentially undisturbed camps inside NNP was therefore added to the sampling frame at the end of round three in November 2022 (Figure 1 and Data S1 and S2). Each circuit was surveyed over a period of approximately 2 weeks, staying at each camp for two nights before departing for the next camp the following morning. The radial surveys of various detected human, livestock and wild mammal activities around surface water bodies within a 2 km radius of each camp (Section 2.4) were carried out on the mornings after arriving at each camp (Duggan et al. 2024).

The designated protected area of the ILUMA WMA acts as a buffer zone with mixed land cover between the rigorously conserved, essentially intact natural ecosystems of Nyerere National Park (NNP) immediately to the east and the villages and farmland along its western border. Some locations inside nearby parts of NNP were therefore added to the sampling frame (Figure 1, Data S1) as clear‐cut examples of essentially intact natural ecosystems. This overall study area and sampling frame (Figure 1, Data S1) was therefore ideal for comparing different ecosystem integrity survey methods (Sections 2.3 to 2.5) because it spans the full range of *de facto* conservation protection levels. This comprehensive range different land use practices and ecosystem characteristics were also considered ideal for the mosquito ecology investigations reported elsewhere (Walsh 2023; Kavishe et al. 2025) because a wide diversity of mammalian species were known to be present (Duggan et al. 2024) and potentially available for host‐seeking *Anopheles arabiensis* malaria vector mosquitoes to feed upon.

### 2.3 Consensus Perception‐Based Estimation of a *Subjective Natural Ecosystem Integrity Index* (SNEII) Score for Each Camp Visited

Following the three rounds of data collection, but prior to any of the numerical syntheses or statistical analyses of quantitative survey data described in sections 2.4 to 2.7, a *subjective natural ecosystem integrity index* (SNEII) was devised, as a convenient, semi‐quantitative alternative indicator for assessing the balance of intactness versus degradation of the natural ecosystem in the area immediately around each camp. This index, based on the holistic impressions of the investigators responsible for carrying out all the field work, was scaled as a percentage score, ranging from 0% to 100%. A score of zero represents a fully domesticated camp, where human settlement has long been established and associated livelihood‐related resource utilization activities, such as land clearing, agriculture, and other resource extraction practices such as charcoal burning are ubiquitous and intensive. At the other end of the scale, a score of 100% represents a fully intact natural ecosystem, with no evidence of human disturbance of any kind that was apparent to the investigators who visited the area. Individual scores were carefully assigned based on a consensus reached through discussions among the three investigators on the field team (LD, KW and LT), based on their subjective recollections. Field notes that were recorded during the study and any photographs that were taken were also carefully considered during these discussions. Due consideration was also given to the time of year a camp was visited and the frequency with which it was surveyed because seasonality influences the activities of both humans and animals and can either hinder or enhance the detectability of tracks, spoor and other signs of those activities: For example, rainfall can limit accessibility and visibility while also softening substrates so that they take clear footprints. These consensus SNEII scores were agreed in December 2022, immediately after data collection was completed. The list of scores for each camp was then drafted in *Excel*, discussed a little further among the investigators, and finalized in the format provided in Data S2. Note that this process was completed before the statistical analyses (Section 2.5) of the formally planned quantitative survey data was initiated, to avoid biasing the former toward the latter.

The primary and most important criterion for assigning an ecosystem integrity score was the subjective impressions of the intensity of land degradation caused by the conversion of land for agriculture, deforestation for the purposes of charcoal production, timber harvesting or human settlement and for livestock herding. The intensity of degradation of the land surrounding a 2 km radius of the camp was determined by estimating the proportion of the natural ecosystem remaining more‐or‐less intact, based on informal personal observations that were made on an unstructured and unplanned basis while present in the camp and during camp radial surveys. This subjectively estimated proportional intactness of natural land cover was then used as a reference point for initiating the discussions that concluded with the assignment of the score. For example, a camp with an estimated 10% of land that was considered ecologically intact was initially given an approximate score of 10. Camps that evidently belonged on the extreme ends of the scale were assigned first, to provide clear reference points, before those that were less clear and required a more in depth‐discussion. The majority of camps that required extra discussion were areas where intermittent patches of deforestation, small‐scale agriculture and human settlement existed in among expanses of intact woodland or forest. These were all located inside ILUMA WMA, in areas that were either regenerating from previous encroachment, or beginning to experience an influx of human activities.

The investigators' recollections regarding the abundance of wildlife and humans as indicators of ecosystem integrity featured less in conversation when assigning scores but were nevertheless useful as secondary factors to be considered following the discussion of the intensity of land degradation. Recollections of the investigators personal observations clearly indicated that species diversity generally increased with intact natural land cover and that some species, such as bushpig were found more often than others in areas where the natural land cover had been partially disturbed but not entirely converted to agriculture. These criteria were useful when deciphering the scores of multiple camps within ILUMA that seemed to have similar levels of intact woodland or forest and degradation. It also proved useful for areas of regenerating woodland that had been previously cleared by humans. Regenerated woodland where the investigators remembered seeing several different species of wild mammal diversity tended to result in a higher ecosystem integrity score being assigned, whereas woodlands in the early stages of regeneration that appeared to have low wildlife diversity were generally assigned lower ecosystem integrity score. However, considerations of perceived wildlife population characteristics as an indicator of ecosystem integrity proved somewhat complex and uncertain, as wildlife communities' composition and diversity naturally change with historical and actual land cover. The ability to distinguish historical and current land cover types was also an important factor when assigning the values for this index, as it was vital to not mistake natural grasslands, scrublands, and valleys for areas that had been deforested.

Based on subjective impressions, it seemed clear that the presence of humans was usually negatively associated with natural ecosystem integrity. However, it was not a consistently reliable criterion as some human activities had little to no impact on their surrounding environment. For example, small, legal fishing camps along the banks of the Kilombero river in the north of ILUMA (camp numbers 14, 15 and 26) are long‐established settlements but were nevertheless assigned high ecosystem integrity scores (Duggan 2023; Duggan et al. 2024).

Although meat and fish poaching were not perceived to be noticeably associated with direct effects on ecosystem integrity, any evidence of these illegal activities nevertheless led to the investigators assigning slightly lower SNEII scores, depending on the intensity observed. After discussion and the initial list of SNEII values was drafted, the scores were reviewed again to ensure that the order and distribution of the natural ecosystem integrity index scores were assigned as well as possible regarding the above criteria. No two camps were assigned exactly the same score and the differences between assigned scores intended to be reasonably representative of the magnitude of their perceived *de facto* differences. Final assigned scores for the SNEII at each camp are detailed in Data S2.

### 2.4 Quantitative Radial Surveys of Human, Livestock and Wild Mammal Activities Around Surface Water Bodies

**FIGURE 2:**
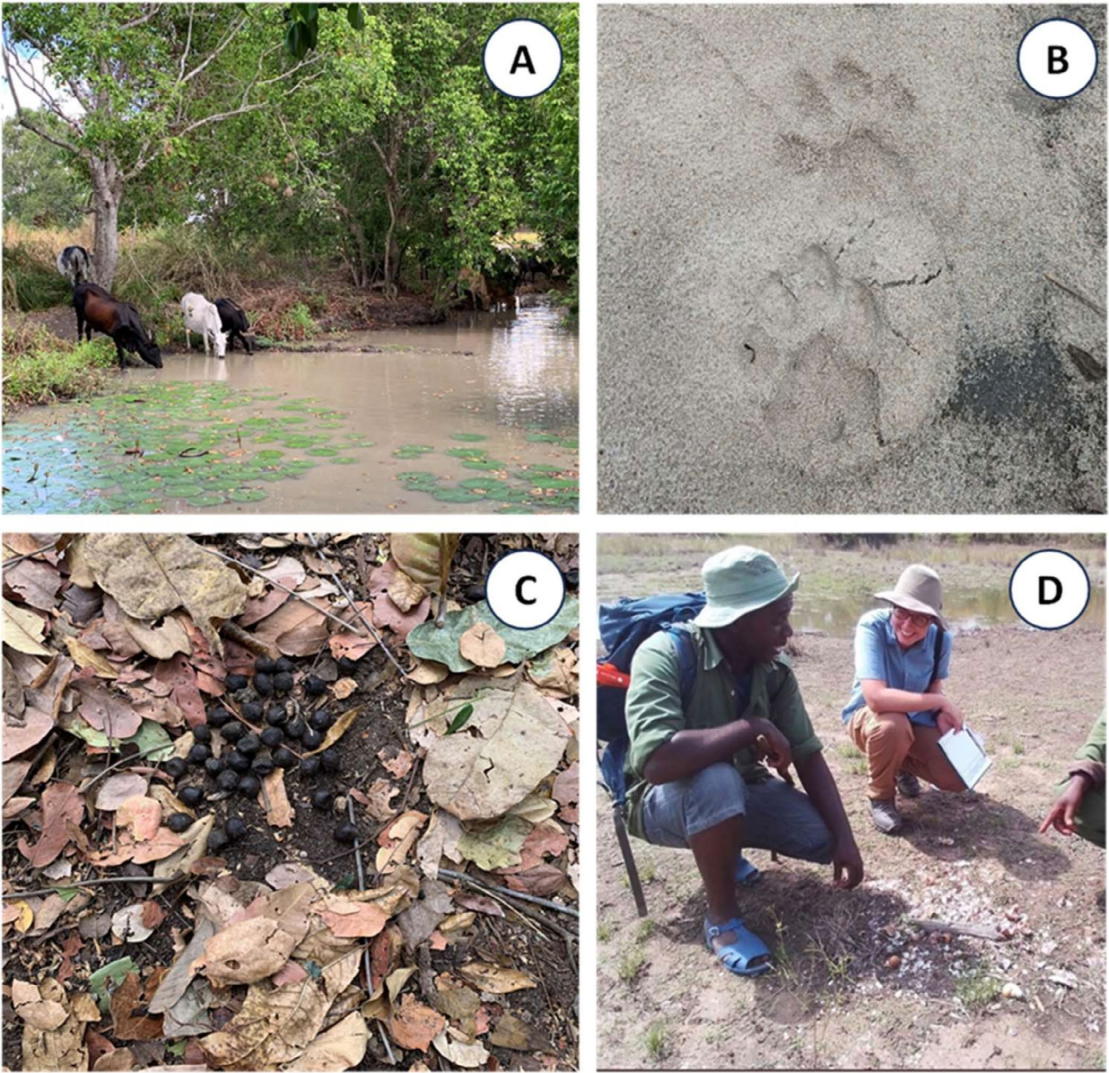
Photographs illustrating different ways in which signs of livestock, humans or wild mammals were detected and recorded as either direct sighting, tracks, spoor or other signs. (A) Illustrates a typical direct sighting of cattle. (B) Illustrates an example of a rare observation, namely fresh lion prints on damp sand that resulted in perfectly clear footprints and an unambiguous identification of the animal and the age of its tracks. In contrast, (C) illustrates a typical example of one of the most frequently recorded detections throughout the entire survey‐Hartebeest spoor, in this case approximately 3 days old. (D) illustrates a particularly common example of “other signs”, specifically broken shells and a stick used as an anvil by water mongoose to crack open snails and shellfish.

As explained in further detail elsewhere (Walsh 2023; Duggan 2023; Duggan et al. 2024) radial surveys for all signs of activity by humans, livestock or wild animals (Figure 2 and Data S3) were carried out along the fringes of water bodies that were first surveyed for mosquito larvae by the accompanying investigators focusing on malaria vector ecology, so the exact direction taken and waterbodies surveyed was primarily determined by the requirements of those entomological surveys. These radial surveys of animal activity around each camp location (Figure 1, Data S1) included all waterbodies within a 2 km radius of that camp that could be surveyed within a maximum of 4 h. Such surveyed water bodies included seasonal streambeds and the pools of water within them, puddles and pools outside of streambeds, waterholes, pools of water in rice fields and rivers and any other surface water body that might attract mosquitoes, people or animals, even if they were clearly ephemeral in nature.

All the survey tools used to collect these data are provided in Data S4–, S7, and the data themselves in Data S8. Note that although the data relating to various recorded signs of various human natural resource utilization practices (Data S3, Figure S3.1–.2 and Data S5, section 1X) and land cover attributes (Data S3, Figure S3.3 and Data S5, section 3X), as well as different signs of activity by humans and livestock (Data S3, Figure 2A and Data S5, subsections 21 and 22), were not reported in the previous study that describes these field procedures in detail, because they were not directly relevant (Duggan et al. 2024), they are reported herein and included in Data S8.

### 2.5 Numerical Estimation of Various Conventional Indices of Wild Animal Community or Whole Ecosystem Integrity Based on Quantitative Data From the Radial Activity Surveys

Estimates for each of the following indices of the integrity of wild animal communities or the whole ecosystem at each camp were calculated as follows based on the data obtained through radial activity surveys (Section 2.4) and presented in Data S2. All the following analyses were carried out using the *R* open‐source software package through the *RStudio* version 2023.03.0 + 386 environment. While only data relating to activities by wild mammals was used to calculate scores for the first three indices of wild animal community integrity for each camp, the fourth and final index described below, intended as an indicator of whole ecosystem integrity, included all of the variables available in Data S8. Assigned scores for the each of the four following indices at each camp are detailed in Data S2.

#### 2.5.1 Species Richness Index (SRI)

This first indicator of wild animal community integrity was chosen due to its conceptual simplicity, calculated as the absolute number of species in a given area (Whittaker, Willis and Field 2001). Estimates of the Species Richness Index (SRI) were calculated for each camp based on wildlife activity data only, using the *plyr* package and *richness* command. SRI is calculated as: $\sum_{i=1}^{x}n$, where *n* = the number of species detected (Moore 2013), with higher values indicating higher biodiversity.

#### 2.5.2 Simpson's Index of Diversity (SID)

Simpson's Index of Diversity was preferred to Shannon's index because estimates for the latter are more strongly influenced by species richness, while the former is more heavily influenced by evenness (Alatalo 1981). Also, the SRI already takes richness into account, and the evenness characteristic of SID may reveal even slight differences between locations (DeJong 1975). Values for SID range from zero (no diversity) to one (highest possible diversity, and are calculated as 1‐ D, where D = $\sum_{i=0}^{x}\mathrm{iPi}$ ^2^, where P_i_ (P_i_ = $\frac{\mathrm{Xi}}{N}$)) is the proportion of observations accounted for by each species *i*, *x*_*i*_ is the total number of each species recorded and N is the total number of individuals recorded.

#### 2.5.3 Objective Wild Animal Community Integrity Index (OWACII)

The data reduction analysis used to generate an objective index of wild animal community integrity was carried out using the *factoextra* package. Like a range of similar composite indicators from a diversity ecological studies (James and McCulloch 1990; Ortega et al. 2004; Castela, Ferreira, and Graça 2008; Reza and Abdullah 2011; Janžekovič and Novak 2012; Caniani et al. 2016) OWACII represents a synthetic statistical summary parameter, based on principal component analysis (PCA) of values for all the various types of detections of all the wildlife species for each camp in the processed datasets provided in Data S8. Scores obtained from the PCA range from −8.8, where the lowest number of detections of different types of direct sighting, tracks, spoor, and other signs of wildlife activity was recorded, to 30.1 where the highest numbers were observed. To make these values easier to interpret, they were normalized by scaling as their Z‐score means.

#### 2.5.4 Objective Natural Ecosystem Integrity Index (ONEII)

Synthetic statistical estimates of this more comprehensive index of overall ecosystem integrity, were again estimated using the *factoextra* package to obtain a PCA‐based index (James and McCulloch 1990; Ortega et al. 2004; Castela, Ferreira, and Graça 2008; Reza and Abdullah 2011; Janžekovič and Novak 2012; Caniani et al. 2016) of all the recorded detections of wildlife, livestock, and humans, and also the summary indicators of land cover at each camp. This index combines all the recorded indicators of wildness with those of encroachment to provide a composite score for comparing the ecological status of different camps. Values range from 87.7, which was the lowest score from a location with almost fully domesticated land cover and minimal signs of wildlife activity, to −13.6 at one of the camps with the highest levels of wildlife activity and the lowest levels of human activity. Again, to make these values easier to interpret, they were scaled as their Z‐score means.

### 2.6 Comparison of Novel Subjective Natural Ecosystem Integrity Index (SNEII) With all Four Objectively Estimated Indicators of Wild Animal Community or Whole Ecosystem Integrity

Again, analysis was carried out in *R* with *RStudio*, this time using the *ggpbr* package while *ggplot2* and *scales* were used for graphing. All maps were drafted using QGIS version 3.28.2., with built in layers from OpenStreetMap and Google Maps in the XYZ Tiles pane, and shapefiles of the study area created using collected GPS coordinates. In order to test the utility of the novel SNEII, correlations between the camp scores obtained for it and for each of the four indices estimated as described in Section 2.5 were assessed in a non‐parametric manner using Spearman's ranked correlation test.

### 2.7 Comparing the Sensitivity of the Subjective Natural Ecosystem Integrity Index (SNEII) to Various Observed Human Activities With That for Objective Alternative Indices

In order to determine how sensitive the SNEII was to various observed human activities, and compare its responsiveness to them, with those of all three alternative objective, quantitative indices of wild animal community integrity (SRI, SID and OWACII), generalized linear modeling (GLM) analysis was undertaken for each index individually as alternative dependent variables. Note, however, that such GLM analysis was not carried out on ONEII because variables relating to human activity were used in creating this composite index of the integrity of the natural ecosystem as a whole, so testing for its dependence upon the same activities that were used to derive it would be confounded by circular logic. Each human activity was firstly scaled using Z‐score means, using the same calculation method described in section 2.5. All analyses were then carried out in *R* with *RStudio*, using the *hist* and *lme4* packages.

First histograms of the distribution of scores for the wild animal community integrity index plotted, to determine whether it was normally distributed or might require log or inverse transformations to normalize. Next, a series of univariate GLMs for each dependent variable index were fitted defining SRI as the dependent variable and one given human activity as the sole independent variable. Multiple distributions and link functions were specified until the best fit model with the lowest AIC score was identified. Multivariate analyses were then carried out using forward stepwise selection procedure to build the most informative and parsimonious explanatory model possible, beginning with the human activity variable with the lowest P value in univariate analysis. Human activities were added and removed from the model based on standard objective criteria specified a priori (See footnotes to Tables S10.1–S10.3) until the best fit model based on AIC score was identified: each retained variable was required to at least approach significance in the model it was included in and significantly improve the goodness of fit relative to the otherwise equivalent model from which it was excluded. The results of both univariate and multivariate analyses on species richness index are presented in Data S10.

## 3 Results

Informal observations of the three investigators who assigned these SNEII scores indicate that initial broad consensus regarding the ranking of locations, and then assignment to approximate quantiles of 10% (equivalent to a simpler scale of 1–10) was remarkably easy to reach, but that the finer distinctions between similar individual locations took a little more time. On no occasion did any of the three investigators diverge substantively from the other two on that initial approximate scoring, and most of the lengthier second round of discussions was spent resolving exact percentage scores within a maximum range of 5%.

**FIGURE 3:**
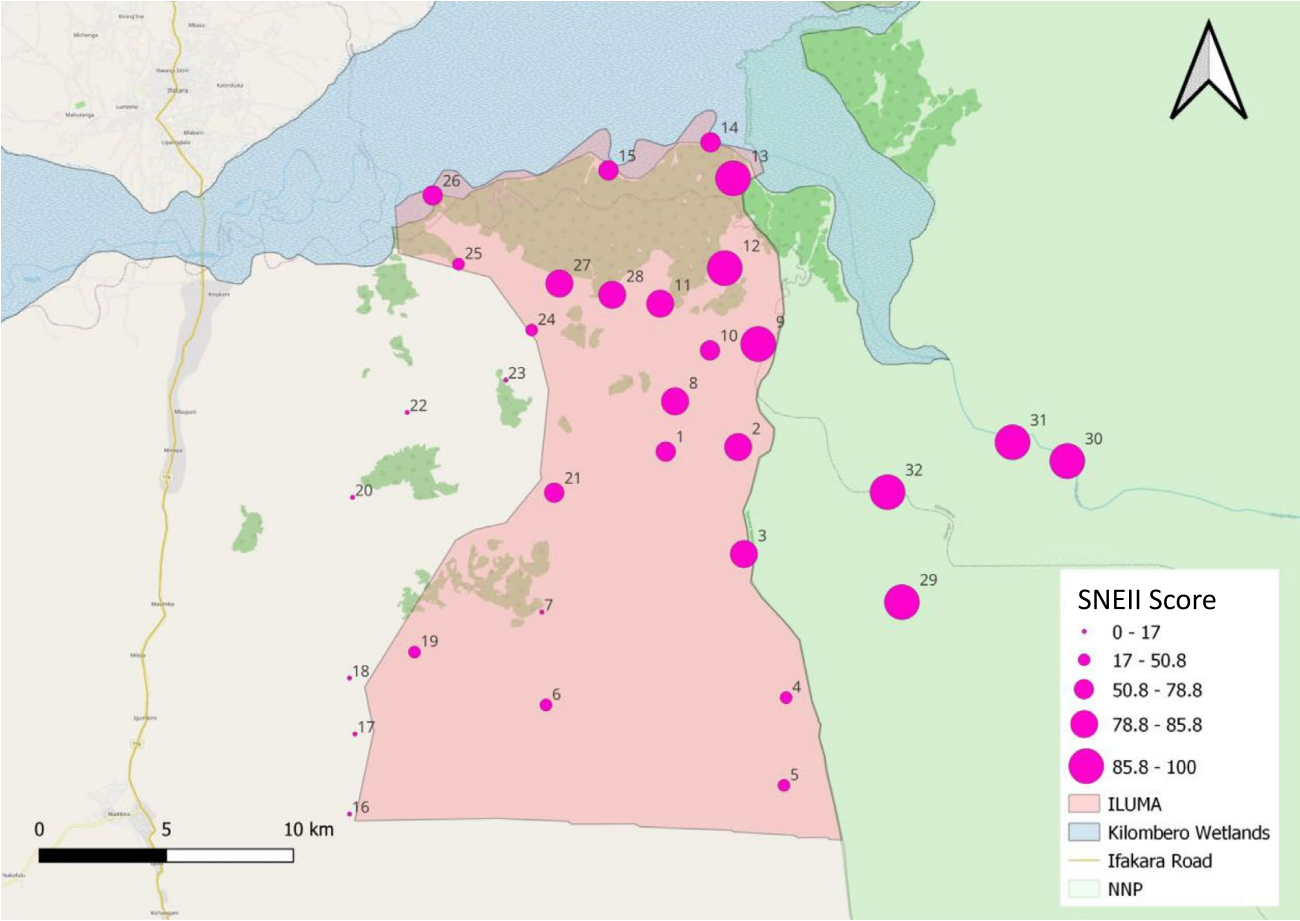
Map of the ILUMA WMA, displaying the Subjective Natural Ecosystem Integrity Index (SNEII) scores estimated for each of the 32 camps described in Data S2, as described in Section 2.4.

The SNEII scores obtained for each camp clearly indicated that while the north‐east corner of the ILUMA WMA and areas stretching westwards and southward, along the Kilombero River and the boundary with NNP, were generally quite intact natural land cover, the integrity of the natural ecosystem had been substantially compromised across most of the rest of the WMA (Figure 3). Reassuringly, the cartographic picture painted by the SNEII was overwhelmingly consistent with those obtained with each of the objective alternatives (Data S9) based on laborious formal quantitative surveys of human, livestock and wildlife activity (Section 4.4) and expert statistical analysis (Section 4.5).

**FIGURE 4:**
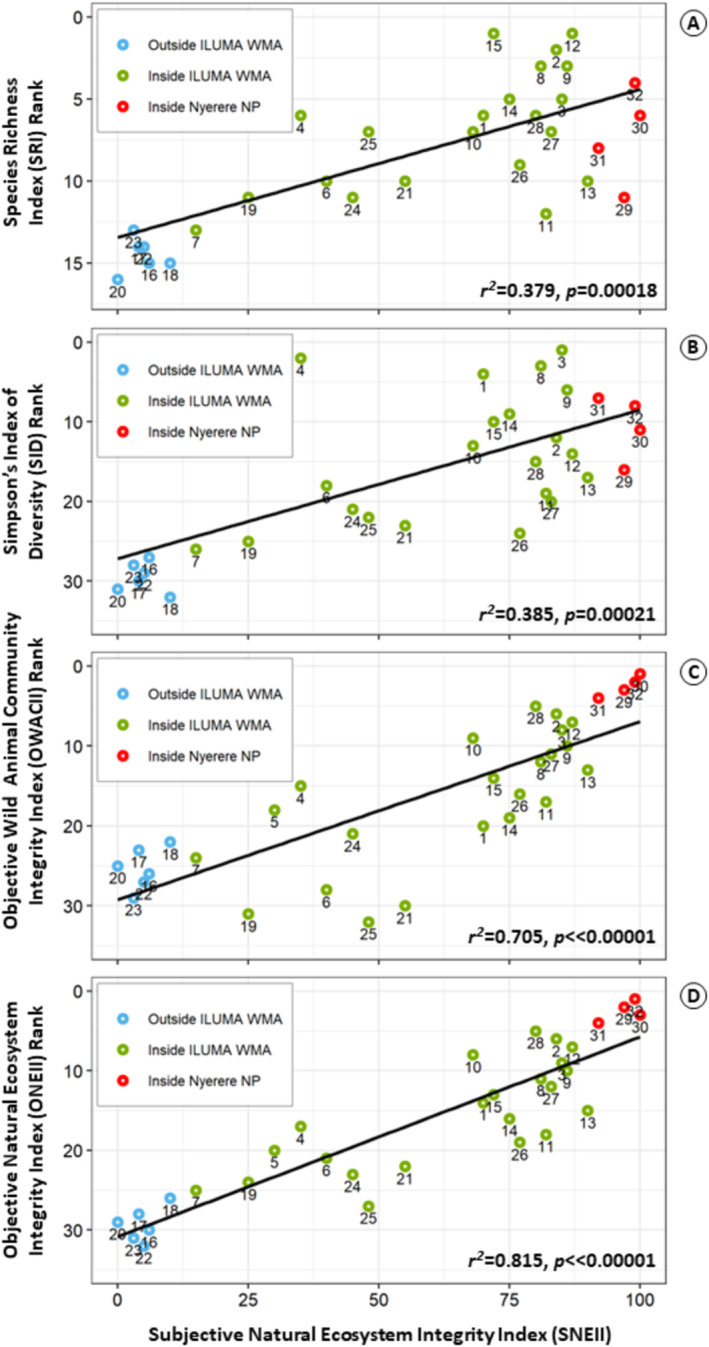
Plots of the correlations observed between assigned subjective natural ecosystem integrity (SNEII) scores for each camp and each of four alternative objective indices of wild animal community or natural ecosystem integrity that were estimated from objective analysis of formal quantitative radial surveys of wildlife, livestock and human activities at the same camp. Each point represents a numbered camp and color denotes its location, with (A, B, C and D) plotting the Species Richness Index (SRI), Simpsons Index of Diversity (SID), Objective Wild Animal Community Index (OWACI) and Objective Natural Ecosystem Integrity Index (ONEII) values estimated as described in Sections 2.4 and 2.5 against the SNEII values assigned as described in Section 2.3.

Furthermore, SNEII scores correlated strongly with all four of the objective indicators of wild animal community or whole natural ecosystem integrity that were estimated directly from formally collected quantitative survey data by the same investigators at the same locations (Figure 4). When comparing SNEII and ONEII it is important to note that the scores for ONEII run from positive to negative, with the most negative value representing the highest ranked camp. Although only a small handful of camps have been awarded the exact same rank by SNEII and any of the four objective alternatives, most ranks for most camps were within a few places of each other for all four objective alternatives (Data S2).

Perhaps unsurprisingly, SNEII correlated most strongly with the ONEII (Figure 4), which was the closest of the four objective alternatives to it in terms of its holistic intended meaning and purpose: Not only did the estimated ONEII scores consider indicators of wild animal diversity and activity levels, they also incorporated indicators for a comprehensive range of human and livestock activities, as well as several land cover attributes (Data S3–, S6). Having said that, the WACII came a close second, while the correlations with SRI and SID were much weaker (Figure 4). Indeed, many of the most ecologically undisturbed camps in the WMA ranked higher in terms SRI and SID than even the 5 camps inside NNP (Figure 4 and Data S2). It therefore appears that SNEII, and also the more laborious but objective ONEII, may be more useful holistic indicators of ecosystem integrity per se than the simpler SRI and SID, especially across study areas with mixed natural land cover types, each with their own distinctive species communities and ecosystem characteristics.

While it is not surprising that the four camps sampled in NNP had the highest SNEII scores, it is nonetheless notable that the 9 next‐best camps located within neighboring WMA all scored 80% or better (Data S2). This indicates that in parts of ILUMA that are well protected, human disturbance remains low and the ecosystem is largely intact. However, SNEII scores were very low on either side of the WMA's western border and all six neighboring villages along that boundary were assigned the lowest scores of 10% or less (Figure 3 and Data S2, camps 16, 17, 18, 20, 22 and 23). The other camps inside ILUMA with low SNEII scores all exhibited some degree of human encroachment that was immediately obvious to the investigators during their visits: For example, camps 7 and 15 both have extensive rice agriculture, while camp 7 also had substantial human settlement. Nevertheless, close examination of Data S2 suggests that human settlement per se does not necessarily result in ecosystem degradation: The permitted fishing camps established along the south bank of the Kilombero River, specifically camps 14, 15 and 26 in Figure 3, have respective scores of 75%, 72% and 77%, respectively, despite being *de facto* villages.

Beyond providing such reassuringly sensible descriptive insights, the SNEII also proved to be far more statistically sensitive to variations in observed frequencies of human activities (Table 1) than any of the three composite objective indices of wild animal community integrity estimated from formally surveyed quantitative indicators (Data S10, Tables S10.1–S10.3). While all three objective alternatives were significantly associated with livestock herding, no other associations could be detected for SRI, whereas SID and OWACII could only be associated with one other human activity each by multivariate regression, namely fishing and meat poaching, respectively. In stark contrast, multivariate analysis indicates SNEII was sensitive to no less than six distinct human activities, namely livestock herding, charcoal burning, timber harvesting, settlement rice farming and other tillage farming.

**TABLE 1:** Results of univariate and multivariate generalized linear modeling analyses of scaled human activity indicators as determinants of the estimated Subjective Natural Ecosystem Integrity Index (SNEII) for each camp surveyed, with statistically significant associations highlighted in bold. The best fit models identified for this SNEII outcome all assumed a Gaussian distribution and identity link function for the dependent variable.

Activity	Univariate			Multivariate		
	*β* **±** SEM	*t*	*p*	*β* **±** SEM	*t*	*p*
Livestock Herding	**−29.3 ± 3.1**	**−9.51**	**<< 0.0001**	**−17.0 ± 2.9**	**−5.85**	**<< 0.0001** ^a^
Charcoal Burning	**−14.5 ± 5.6**	**−2.59**	**0.0146**	**6.8 ± 2.7**	**3.01**	**0.0058** ^a^
Timber Harvesting	−7.7 **±** 6.0	−1.27	0.213	**4.4 ± 2.0**	**2.25**	**0.0333** ^a^
Fishing	1.1 **±** 6.2	0.18	0.862	3.6 **±** 2.4	1.50	0.1454^b^
Hunting	−4.9 **±** 6.1	−0.80	0.429	−0.88 **±** 2.2	−0.40	0.6917^b^
Human Settlement	**−23.3 ± 4.5**	**−5.19**	**<< 0.0001**	**−6.5 ± 2.7**	**−2.38**	**0.0256** ^a^
Well	**−21.4 ± 4.8**	**−4.49**	**0.0001**	3.3 **±** 3.0	1.10	0.2814^b^
Meat Poaching	−8.2 **±** 6.0	−1.37	0.180	−1.2 **±** 2.0	−0.62	0.5388^b^
Human Presence	**−21.6 ± 4.7**	**−4.6**	**0.0001**	5.4 **±** 3.2	1.70	0.1011^b^
Rice Farming	**−27.9 ± 3.5**	**−8.00**	**<< 0.0001**	**−9.4 ± 2.9**	**−3.25**	**0.0033** ^**a**^
Other Tillage Farming	**−29.4 ± 3.0**	**−9.69**	**<< 0.0001**	**−9.7 ± 3.7**	**−2.59**	**0.0157** ^a^

Abbreviation: SEM, standard error of the mean for the *β* coefficient estimate.

^a^ As estimated from the final, most parsimonious best‐fit GLM, in which this was included on the basis of either being a significant (*p* ≤ 0.05) determinant of SNEII or approached significance (*p* ≤ 0.10) as a determinant of SNEII and significantly (*p* ≤ 0.05) improved the goodness of fit based on the estimated Akaike Information Criterion (AIC).

^b^ As estimated from the last GLM in which this variable was still included before being removed on the basis that it was not a significant determinant of the SNEII (*p* > 0.05) or approached significance (*p* ≤ 0.10) but did not significantly improve the goodness of fit based on the estimated AIC (*p* > 0.05).

Interestingly, these detailed regression analyses confirm that the specific livelihood‐associated terrestrial activities of livestock herding, rice farming and other forms of tillage agriculture are all stronger predictors of low SNEII scores than human settlement per se (Table 1). Although the association of charcoal burning and timber harvesting with high SNEII scores (Table 1) might initially seem counter‐intuitive, this is readily explained by reverse causality: Intact forest not only provides the wood required to carry out these activities, it also provides useful cover to those conducting them without a permit.

## 4 Discussion

Overall, SNEII appears to be a reasonable, convenient, holistic and cost‐effective substitute for the four objective alternatives, all of which rely on expert analyses (Section 2.5) of large amounts of laboriously collected formal quantitative survey data (Section 2.4 and Data S3–, S8). As SNEII and ONEII both aimed at combining observations of wildlife, livestock and human activities, together with land cover attributes, to grade each of the camp locations, the reassuringly close correlation between these two conceptually similar indices (Figure 4D) was particularly reassuring with respect to the validity of the former as a potential substitute for the latter. Furthermore, the correlation of SNEII with ONEII (Figure 4D), and to a slightly lesser extent with OWACII (Figure 4C), appear far more robust to variations in natural land cover than simpler indicators of species richness and diversity (Figure 4A,B, respectively). It therefore appears that all three of these more multifactorial indicators may be far more useful than SRI or SID for assessing conservation effectiveness across extensive landscapes encompassing several distinct habitat types, with SNEII and ONEII apparently being the most holistic and broadly useful options.

Beyond being an apparently adequate substitute for all these objective measures, with no obvious comparative disadvantages, the SNEII may well be a more sensitive and powerful indicator of illegal encroachment by people and their livestock within the protected area of the WMA than any of the objective alternatives (Table 1 vs. Data S10). This latter observation is consistent with the experiences of other investigators all across the tropics, who report that use of such subjective, intuitive indicators can provide many insights into ecosystem state and functionality, not to mention conservation intervention opportunities, that would have been missed if they had relied solely upon purely objective, formally planned, quantitative methodologies (Raddaoui 2009; Vergara‐Asenjo, Sharma, and Potvin 2015; Beaudoin et al. 2016). Indeed, beyond yielding more detailed analytical insights into ongoing conservation successes, failures and opportunities in this southern Tanzanian WMA (Duggan 2023), SNEII also proved to be a powerful environmental predictor of the malaria vector mosquito population composition across the same study area (Walsh 2023).

In stark contrast with the SNEII, all four objective alternatives required large amounts of survey data (Section 2.4 and Data S8) and numerical expertise (Section 2.5) to estimate, neither of which may be available, or indeed appropriate, in all contexts where the ecological integrity of an area needs to be assessed quickly, practically and affordably. Indeed, SNEII represents an intuitive approach to expressing consensus subjective perceptions, permitting rapid determination of a location's status in approximate semi‐quantitative terms, without requiring any numerical data or statistical knowledge. Scoring of SNEII requires only that the individuals responsible for scoring this index have reasonable understanding of the ecosystem in question. For example, all three investigators who ranked these camps as described herein, were all bachelors' degree graduates with no prior experience of comparable habitats. Indeed, community‐based professionals with especially relevant in‐depth informal expertise, like the VGS who supported this study, may well prove to be ideal observer‐investigators in more participatory applications of the same approach in due course. If this proves to be the case, it may be broadly useful for larger scale application in sustained, long‐term conservation monitoring, as distinct from time‐limited research projects like this study, because it produces rapid, affordable, practical and meaningful assessments of ecosystem status. One of the greatest advantages of this index is that it produces a holistic composite score based on all recorded parameters and unrecorded personal impressions of the ecosystem that may be readily interpreted on an intuitive basis and may be discussed with a variety of stakeholders in conservation areas, notably in the communities living in or alongside them.

Nevertheless, some important caveats need to be borne in mind when considering whether to adapt this new perception‐based approach to routine programmatic applications. For example, although the field‐based components of this study encompassed all the main seasons routinely experienced in southern Tanzania, it was limited in extent to of a single calendar year. Given the subjective nature of this approach, and the inevitable turnover of individual observers that may be expected over longer periods of longitudinal monitoring, it may well be especially vulnerable to *shifting baseline syndrome* (Soga and Gaston 2018; Alleway et al. 2023). It therefore remains to be seen how well such procedures may work over longer periods, particularly for longitudinal monitoring and evaluation applications.

Furthermore, this approach only captures forms of disturbance that were apparent to the observers, who in this case had more extended opportunities to observe while carrying out the objective radial and transect surveys than would otherwise be the case. It is therefore possible that some of the SNEII scores reported herein somewhat exaggerated the true underlying level of ecological intactness for locations within the study area. It is also plausible that many of the more infrequent or subtle forms of disturbance reported herein might not be observed informally if those laborious formal surveys of human, livestock and wildlife activities were omitted (Duggan 2023; Duggan et al. 2024).

Also, caution may be required before adapting to areas that lack such extremes of land conversion and natural ecosystem integrity as those respectively found in the west and north‐east of this study area (Figure 3). This particular research project included areas ranging from fully domesticated villages and farmland all the way through to fully intact natural ecosystems inside a major national park. The responsible investigators found that the inclusion of such survey sites outside the WMA, at both extremes of the full possible spectrum of natural ecosystem integrity, helped them greatly in assigning a score because they provided obvious gold standards with which locations exhibiting a kaleidoscope of different intermediate characteristics within ILUMA could be intuitively compared. In more homogenous areas, where ecological gradients are less obvious and fully domesticated or ecologically intact areas are lacking, it may prove more difficult to assign reliable scores. Where possible, it may therefore be wise to similarly extend survey sampling frames for other conservation areas slightly beyond their boundaries, to include locations on either end of the converted‐to‐intact spectrum that provide observers with similar points of reference at both extremes.

Also, the most obvious way in which this subjective approach may represent a double‐edged sword is subjectivity itself. Concerns about investigator bias pervade even the supposedly objective quantitative physical and biological sciences (Ioannidis 2005; Sarewitz 2012) and need particularly careful attention in semi‐quantitative and qualitative sociological investigations (Paulhus 1991; Tong, Sainsbury, and Craig 2007; Schumm 2021), wherein the “the researcher is the instrument” (Pezalla, Pettigrew, and Miller‐Day 2012). Further development and assessment of this approach will therefore need to determine whether procedures similar to those described herein can be applied consistently enough across different time periods with different personnel to yield consistent results, even within a given locale and institutional context, so that SNEII estimates are obtained that are comparable enough for meaningful longitudinal monitoring. Furthermore, achieving consistency across lots of different conservation areas, especially independently managed community‐based organizations, to enable pooling of data for evaluation purposes at larger national and regional scales may prove even more challenging, necessitating more formally structured, standardized approaches (Burgmann et al. 2011; Martin et al. 2012; Hemming et al. 2018).

Having said that, it is reassuring that the three relatively inexperienced investigators responsible for assigning the scores experienced negligible difficulty in reaching consensus, suggesting that three scorers are most probably sufficient in most cases. Conveniently, three is the smallest odd number greater than one, allowing consensus to be established around the perceptions of the smallest possible majority in cases where one scorer diverges from two others. Nevertheless, it must be recognized that the perceptions of these three investigators were not formed independently of each other, so this remarkable degree of consensus is unlikely to reflect corresponding levels of certainty. All three scorers were led, informed and influenced by the same team of VGS, so the many shared conversations with these community experts that occurred in the field obviously contributed to such overlapping perceptions.

Also, it should be noted that temporal and spatial comparability within a given administrative unit may nevertheless be sufficient to inform effective management at local scale, which often trumps the priorities of stakeholders at national and international levels (Danielsen, Burgess, and Balmford 2005; Bennun et al. 2005; Poulsen and Luanglath 2005; Setty et al. 2008; Raddaoui 2009; Beaudoin et al. 2016; Vergara‐Asenjo, Sharma, and Potvin 2015; Evans, Guariguata, and Brancalion 2018; Kusters et al. 2018; Thiao et al. 2019). Furthermore, data management and analysis methods for integrating such locally variable monitoring data at these higher levels are improving and becoming more responsive to such bottom‐up management and evaluation strategies (Bennun et al. 2005; Raddaoui 2009; Beaudoin et al. 2016; Vergara‐Asenjo, Sharma, and Potvin 2015; Evans, Guariguata, and Brancalion 2018; Kusters et al. 2018; Thiao et al. 2019; Mandeville et al. 2023). Indeed, it is particularly notable that spatially explicit applications of such subjective, holistic approaches to landscape assessment, often referred to as participatory mapping, have repeatedly proven complementary, and sometimes superior, to purely physical remote sensing methods (Beaudoin et al. 2016; Raddaoui 2009; Vergara‐Asenjo, Sharma, and Potvin 2015). Simplification of the scoring system to Likert scales may be useful for adaptations to community‐based applications, possibly enabling relatively straightforward quality control and assurance without needing any advanced statistics, particularly for new members of observer teams in long‐term monitoring programmes.

Note also, however, that the subjective nature of this approach, combined with the variable perceptions of individuals, especially community members distributed across diverse stakeholder groups with quite distinct interests, may present as many opportunities as it does challenges. For example, herders, farmers and tourist guides may see the landscape in very different ways, so such semi‐quantitative, consensus‐based approaches to soliciting perceptions from focus groups might be usefully adapted to comparing their distinct views on ecosystem functionality. Such approaches could not only be complemented by conventional qualitative procedures like focus group discussions and in‐depth interviews but also by participatory methods for soliciting community perspectives in photographic formats (Wang and Burris 1997; Wang 1999) that have, in our experience, proven invaluable for public health applications in similar contexts (Makungu et al. 2017; Damus et al. 2024). Indeed, “innovation is needed to further develop culturally relevant citizen science that benefits participants and end users” (Pocock et al. 2019), particularly in low‐income rural contests across the tropics, where conservation efforts need to also augment community livelihoods in fragile local economies.

## 5 Conclusion

The subjective, perception‐based approach described herein to score ecosystem integrity at defined locations in terms of SNEII performed well in comparison with all four objective alternatives based on extended expert analyses of laboriously collected formal quantitative survey data. As explained in further depth elsewhere, regression analyses using the SNEII as the dependent variable yielded far more detailed analytical insights into ongoing conservation challenges than any of these objective alternatives (Duggan 2023), as well as the population dynamics of malaria vector mosquitoes feeding on various human and animal hosts across the study area (Walsh 2023; Walsh et al. 2024). The novel SNEII approach described herein may therefore offer a powerful and scalable new option for routine ecosystem monitoring in the future, because it is simple and affordable and can be readily applied by the VGS or similar community‐based personnel during regular patrols, or even by amateur local community members, using participatory citizen science approaches. It might also be adapted to assessing the distinct perspectives of different stakeholder groups within communities regarding natural resource management and ecosystem functionality across the landscapes they live in.

## Author Contributions

**Lily M. Duggan:** data curation (equal), formal analysis (equal), investigation (equal), writing – original draft (equal), writing – review and editing (equal). **Katrina A. Walsh:** data curation (supporting), formal analysis (equal), methodology (equal), writing – review and editing (supporting). **Lucia J. Tarimo:** data curation (supporting), methodology (supporting). **Deogratius R. Kavishe:** data curation (equal), project administration (equal), resources (equal), writing – review and editing (equal). **Ramiro D. Crego:** writing – review and editing (equal). **Manase Elisa:** writing – review and editing (supporting). **Fidelma Butler:** supervision (equal). **Felister Mombo:** supervision (equal), writing – review and editing (supporting). **Gerry F. Killeen:** conceptualization (equal), formal analysis (equal), funding acquisition (equal), project administration (equal), supervision (equal), writing – review and editing (equal).

## Conflicts of Interest

The authors declare no conflicts of interest.

## Supporting information


**Data S1.** Table S1: The number, name, location, coordinates, and ecological characteristics of each camp location, together with the quadrant circuit to which it was assigned and the number of times it was surveyed (Duggan 2023; Duggan et al. 2024); https://doi.org/10.5281/zenodo.10955565.

**Data S2.** Table S2. Scores for various indices of the integrity of the wild animal community or ecosystem as a whole, estimated for each surveyed camp location (Section 4.5) based on either a consensus approach to synthesizing investigator‐perception (SNEII, Section 2.3) of through numerical synthesis (SRI, SID, OWACII and ONEII, Section 2.5) of extensive data from formal, quantitative surveys of observable human, livestock and wildlife activities, as well as land cover characteristics (Section 4.5 and Data S8); https://doi.org/10.5281/zenodo.10955597.

**Data S3.** Figure S3.1–S3.3: Additional photographs, complementing those presented in Figure 2, to illustrate different ways in which signs of livestock, humans or wild mammal activity, as well as land cover attributes, were observed and classified; https://doi.org/10.5281/zenodo.12795058.

**Data S4.** Complete list of all wild herbivores, wild carnivores, wild primates and prosimians and wild rodents sampled in this study; https://doi.org/10.5281/zenodo.10955623.

**Data S5.** Data collection key devised during preliminary study in October and November 2021 and used throughout the course of the field study to inform accurate and consistent recording of data; https://doi.org/10.5281/zenodo.10955638.

**Data S6.** Criteria defining terms used in data dictionary key; https://doi.org/10.5281/zenodo.10955643.

**Data S7.** Data collection sheet developed during preliminary investigation in October and November 2021 and used for all data collection in both transect segment surveys and camp radial surveys throughout the course of the study https://doi.org/10.5281/zenodo.10955725.

**Data S8.** The full data set used for the analysis presented herein; https://doi.org/10.5281/zenodo.12794932.

**Data S9.** Figure S9.1–S9.4: Maps of the ILUMA WMA, respectively displaying the Species Richness Index (SRI), Simpson’s Index of Diversity (SID), Objective Wild Animal Community Integrity Index (OWACII) and Objective Natural Ecosystem Integrity Index (ONEII) scores estimated for each camp as described in sections 2.4 and 2.5; https://doi.org/10.5281/zenodo.10955759.

**Data S10.** Table S10.1–S10.3; Results of univariate and multivariate generalized linear modeling analyses of scaled human activity indicators as respective determinants of the estimated Species Richness Index (SRI), Simpson’s Index of Diversity (SID) and Objective Wild Animal Community Integrity Index (OWACII) for each camp surveyed; https://doi.org/10.5281/zenodo.10955769.

## Data Availability

**Data S1** Table S1: The number, name, location, coordinates, and ecological characteristics of each camp location, together with the quadrant circuit to which it was assigned and the number of times it was surveyed (Duggan 2023; Duggan et al. 2024); https://doi.org/10.5281/zenodo.10955565. **Data S2** Table S2. Scores for various indices of the integrity of the wild animal community or ecosystem as a whole, estimated for each surveyed camp location (Section 4.5) based on either a consensus approach to synthesizing investigator‐perception (SNEII, Section 2.3) of through numerical synthesis (SRI, SID, OWACII and ONEII, Section 2.5) of extensive data from formal, quantitative surveys of observable human, livestock and wildlife activities, as well as land cover characteristics (Section 4.5 and Data S8); https://doi.org/10.5281/zenodo.10955597. **Data S3** Figure S3.1–S3.3: Additional photographs, complementing those presented in Figure 2, to illustrate different ways in which signs of livestock, humans or wild mammal activity, as well as land cover attributes, were observed and classified; https://doi.org/10.5281/zenodo.12795058. **Data S4** Complete list of all wild herbivores, wild carnivores, wild primates and prosimians and wild rodents sampled in this study; https://doi.org/10.5281/zenodo.10955623. **Data S5** Data collection key devised during preliminary study in October and November 2021 and used throughout the course of the field study to inform accurate and consistent recording of data; https://doi.org/10.5281/zenodo.10955638. **Data S6** Criteria defining terms used in data dictionary key; https://doi.org/10.5281/zenodo.10955643. **Data S7** Data collection sheet developed during preliminary investigation in October and November 2021 and used for all data collection in both transect segment surveys and camp radial surveys throughout the course of the study https://doi.org/10.5281/zenodo.10955725. **Data S8** The full data set used for the analysis presented herein; https://doi.org/10.5281/zenodo.12794932. **Data S9** Figure S9.1–S9.4: Maps of the ILUMA WMA, respectively displaying the Species Richness Index (SRI), Simpson's Index of Diversity (SID), Objective Wild Animal Community Integrity Index (OWACII) and Objective Natural Ecosystem Integrity Index (ONEII) scores estimated for each camp as described in sections 2.4 and 2.5; https://doi.org/10.5281/zenodo.10955759. **Data S10** Table S10.1–S10.3; Results of univariate and multivariate generalized linear modeling analyses of scaled human activity indicators as respective determinants of the estimated Species Richness Index (SRI), Simpson's Index of Diversity (SID) and Objective Wild Animal Community Integrity Index (OWACII) for each camp surveyed; https://doi.org/10.5281/zenodo.10955769.
