# Supplementary material for: A Subjective and Intuitive Approach to Rapid, Holistic Assessment of Natural Ecosystem Integrity Across a Community‐Managed Conservation Area in Southern Tanzania
Source: Ecol Evol. 2025 Mar 2;15(3):e70872. doi: 10.1002/ece3.70872 (PMC11872596; doi:10.1002/ece3.70872)
Supplement: Supplementary file 1 — Data S1. Table S1: The number, name, location, coordinates, and ecological characteristics of each camp location, together with the quadrant circuit to which it was assigned and the number of times it was surveyed (Duggan 2023; Duggan et al. 2024); https://doi.org/10.5281/zenodo.10955565. [file ECE3-15-e70872-s003.docx]

**Supplementary Table S1.** The number, name, location, coordinates, and ecological characteristics of each camp location, together with the quadrant circuit to which it was assigned and the number of times it was surveyed.

| **Camp Number** | **Camp Name** | **Circuit** | **Camp Location** | **Camp Coordinates** | **Number of times surveyed** | **Habitat Type** |
| --- | --- | --- | --- | --- | --- | --- |
| 1 | Msakamba | Southeast | Inside ILUMA WMA | -8.25981S, 36.85936E | 3 | Recovering miombo woodland along a small seasonal stream bed. |
| 2 | Msiba wa Deo | Southeast | Inside ILUMA WMA | -8.25821S, 36.88524E | 3 | Intact miombo woodland along a small seasonal stream bed. |
| 3 | Bwawa la Nyati | Southeast | Inside ILUMA WMA | -8.29598S, 36.88732E | 3 | Intact miombo woodland surrounding a large waterhole. |
| 4 | Bwawa la Nandete | Southeast | Inside ILUMA WMA | -8.3467S, 36.9024E | 3 | Degraded miombo woodland surrounding a large waterhole. |
| 5 | Korongo la Bundu | Southeast | Inside ILUMA WMA | -8.37768S, 36.90163E | 3 | Degraded miombo woodland along a small seasonal stream bed. |
| 6 | Bwawa la Namamba | Southeast | Inside ILUMA WMA | -8.34931S, 36.81653E | 3 | Degraded miombo woodland surrounding a large waterhole. |
| 7 | Bwawa la Chakacheni | Southeast | Inside ILUMA WMA | -8.31649S, 36.81507E | 3 | Degraded miombo woodland surrounding a large waterhole. |
| 8 | Bwawa la Njuju | Northeast | Inside ILUMA WMA | -8.24204S, 36.86268E | 3 | Intact miombo woodland surrounding a large waterhole. |
| 9 | Bwawa la Chamvi | Northeast | Inside ILUMA WMA | -8.22176S, 36.89241E | 2 | Intact miombo woodland surrounding a large waterhole. |
| 10 | Bwawa la Miembeni | Northeast | Inside ILUMA WMA | -8.22401S, 36.87518E | 1 | Mostly intact miombo woodland surrounding a waterhole. |
| 11 | Kisima cha Seba | Northeast | Inside ILUMA WMA | -8.20749S, 36.85738E | 2 | Intact miombo woodland adjacent to a small waterhole. |
| 12 | Bwawa la Maya | Northeast | Inside ILUMA WMA | -8.19492S, 36.88045E | 3 | Transition zone between miombo woodland and groundwater forest surrounding a large waterhole. |
| 13 | Bwawa la Mrope | Northeast | Inside ILUMA WMA | -8.16321S, 36.88341E | 2 | Dense groundwater forest surrounding a large waterhole. |
| 14 | Mikeregembe | Northeast | Inside ILUMA WMA | -8.15048S, 36.87539E | 3 | Authorised fishing camp on the banks of the Kilombero river, open but bordering groundwater forest. |
| 15 | Mdalangwila | Northeast | Inside ILUMA WMA | -8.16041S, 36.83887E | 3 | Authorised fishing camp on the banks of the Kilombero river, open but bordering groundwater forest. |
| 16 | Bwawa la Muamachi | Southwest | Village outside western boundary of ILUMA WMA | -8.38785S, 36.74629E | 3 | Village outside the conservation area surrounding a large waterhole. |
| 17 | Tuliza Moyo | Southwest | Village outside western boundary of ILUMA WMA | -8.35954S, 36.74821E | 3 | Village outside the conservation area along a small seasonal stream bed. |
| 18 | Mavimba Porini | Southwest | Village outside western boundary of ILUMA WMA | -8.33982S, 36.74628E | 3 | Village outside the conservation area along a large river and surrounding a large waterhole. |
| 19 | Bwawa la Selesussi | Southwest | Inside ILUMA WMA | -8.33064S, 36.76951E | 3 | Degraded miombo woodland surrounding a large waterhole. |
| 20 | Makingi | Southwest | Village outside western boundary of ILUMA WMA | -8.27593S, 36.74737E | 3 | Village outside the conservation area along a small seasonal stream bed. |
| 21 | Bwawa la Mpunga | Southwest | Inside ILUMA WMA | -8.27426S, 36.81946E | 3 | Highly degraded miombo woodland surrounding a large waterhole. |
| 22 | Kisaki | Northwest | Village outside western boundary of ILUMA WMA | -8.24595S, 36.7669E | 2 | Largest human settlement, at the base of a large hill adjacent to a spring and large waterhole. |
| 23 | Uwanja wa Ndege | Northwest | Village outside western boundary of ILUMA WMA | -8.23448S, 36.80218E | 2 | Human settlement along a small seasonal river. |
| 24 | Bwawa la Mkwajuni | Northwest | Inside ILUMA WMA | -8.21681S, 36.81141E | 2 | Intact miombo woodland surrounding a degraded large waterhole. |
| 25 | Bwawa la Mamba Luhogi | Northwest | Inside ILUMA WMA | -8.19352S, 36.78527E | 2 | Intact miombo woodland surrounding a highly degraded large waterhole. |
| 26 | Funga | Northwest | Inside ILUMA WMA | -8.16924S, 36.776E | 2 | Authorised fishing camp on the banks of the Kilombero river, open but bordering intact groundwater forest. |
| 27 | Bwawa la Mlenda | Northwest | Inside ILUMA WMA | -8.20037S, 36.82132E | 2 | Intact miombo woodland surrounding a large waterhole. |
| 28 | Bwawa la Semka | Northwest | Inside ILUMA WMA | -8.20436S, 36.84023E | 2 | Largely intact groundwater forest surrounding a large waterhole. |
| 29 | Bwawa la Simba | Nyerere | Nyerere NP (East of ILUMA WMA) | -8.31298S, 36.94381E | 1 | Transition zone of mixed miombo woodland and acacia savanna surrounding a waterhole. |
| 30 | Kiboko Zanzibar | Nyerere | Nyerere NP (East of ILUMA WMA) | -8.26313S, 37.00293E | 1 | Open acacia savanna on the banks of the Kilombero river. |
| 31 | Zanzibar | Nyerere | Nyerere NP (East of ILUMA WMA) | -8.2565S, 36.98337E | 1 | Transition zone of mixed miombo woodland and acacia savanna on the banks of the Kilombero river. |
| 32 | Bwawa la Moto | Nyerere | Nyerere NP (East of ILUMA WMA) | -8.2741S, 36.93866E | 1 | Transition zone of mixed miombo woodland and acacia savanna surrounding a waterhole. |
