# Supplementary material for: A Subjective and Intuitive Approach to Rapid, Holistic Assessment of Natural Ecosystem Integrity Across a Community‐Managed Conservation Area in Southern Tanzania
Source: Ecol Evol. 2025 Mar 2;15(3):e70872. doi: 10.1002/ece3.70872 (PMC11872596; doi:10.1002/ece3.70872)
Supplement: Supplementary file 2 — Data S2. Table S2. Scores for various indices of the integrity of the wild animal community or ecosystem as a whole, estimated for each surveyed camp location (Section 4.5) based on either a consensus approach to synthesizing investigator‐perception (SNEII, Section 2.3) of through numerical synthesis (SRI, SID, OWACII and ONEII, Section 2.5) of extensive data from formal, quantitative surveys of observable human, livestock and wildlife activities, as well as land cover characteristics (Section 4.5 and Data S8); https://doi.org/10.5281/zenodo.10955597. [file ECE3-15-e70872-s001.docx]

**Supplementary Table S2.** Scores for of various indices of the integrity of the wild animal community or ecosystem as a whole, estimated for each surveyed camp location (Section 4.5) based on either a consensus approach to synthesizing investigator-perception (SNEII, Section 2.3) of through numerical synthesis (SRI, SID, OWACII and ONEII, Section 2.5) of extensive data from formal, quantitative surveys of observable human, livestock and wildlife activities, as well as land cover characteristics (Section 4.5 and Supplementary file 8).

| Camp | Location | Historic Natural Land Cover | Subjective Natural Ecosystem Integrity Index^a^  (SNEII) | | Species Richness Index^b^  (SRI) | | Simpson’s Index of Diversity^c^  (SID) | | Objective Natural Animal Community Integrity Index^d^  (ONACII) | | Objective Natural Ecosystem Integrity Index^e^  (ONEII) | |
| --- | --- | --- | --- | --- | --- | --- | --- | --- | --- | --- | --- | --- |
|  |  |  | Value | Rank^f^ | Value | Rank^f^ | Value | Rank^f^ | Value | Rank^f^ | Value | Rank^f^ |
| 1: Msakamba | Inside ILUMA WMA | Miombo Woodland | 70 | 17 | 16 | 6 | 0.952 | 4 | -0.507 | 20 | -0.390 | 14 |
| 2: Msiba wa Deo | Inside ILUMA WMA | Miombo Woodland | 84 | 9 | 20 | 2 | 0.934 | 12 | 0.338 | 6 | -0.478 | 6 |
| 3: Bwawa la Nyati | Inside ILUMA WMA | Miombo Woodland | 85 | 8 | 17 | 5 | 0.957 | 1 | 0.250 | 8 | -0.439 | 9 |
| 4: Bwawa la Nandete | Inside ILUMA WMA | Miombo Woodland | 35 | 23 | 16 | 6 | 0.956 | 2 | -0.349 | 15 | -0.370 | 17 |
| 5: Korongo la Bundu | Inside ILUMA WMA | Miombo Woodland | 30 | 24 | 18 | 4 | 0.951 | 5 | -0.468 | 18 | -0.352 | 20 |
| 6: Bwawa la Namamba | Inside ILUMA WMA | Miombo Woodland | 40 | 22 | 12 | 10 | 0.889 | 18 | -0.619 | 28 | -0.347 | 21 |
| 7: Bwawa la Chakacheni | Inside ILUMA WMA | Miombo Woodland | 15 | 26 | 6 | 13 | 0.652 | 26 | -0.594 | 24 | -0.333 | 25 |
| 8: Bwawa la Njuju | Inside ILUMA WMA | Miombo Woodland | 81 | 12 | 19 | 3 | 0.952 | 3 | -0.169 | 12 | -0.411 | 11 |
| 9: Bwawa la Chamvi | Inside ILUMA WMA | Miombo Woodland | 86 | 7 | 19 | 3 | 0.950 | 6 | -0.004 | 10 | -0.418 | 10 |
| 10: Bwawa la Miembeni | Inside ILUMA WMA | Miombo Woodland | 68 | 18 | 15 | 7 | 0.933 | 13 | 0.035 | 9 | -0.443 | 8 |
| 11: Kisima cha Seba | Inside ILUMA WMA | Miombo Woodland | 82 | 11 | 9 | 12 | 0.882 | 19 | -0.410 | 17 | -0.369 | 18 |
| 12: Bwawa la Maya | Inside ILUMA WMA | Miombo Woodland | 87 | 6 | 22 | 1 | 0.928 | 14 | 0.296 | 7 | -0.445 | 7 |
| 13: Bwawa la Mrope | Inside ILUMA WMA | Groundwater Forest | 90 | 5 | 12 | 10 | 0.893 | 17 | -0.244 | 13 | -0.383 | 15 |
| 14: Mikeregembe | Inside ILUMA WMA | Groundwater Forest | 75 | 15 | 17 | 5 | 0.947 | 9 | -0.500 | 19 | -0.382 | 16 |
| 15: Mdalangwila | Inside ILUMA WMA | Groundwater Forest | 72 | 16 | 22 | 1 | 0.941 | 10 | -0.260 | 14 | -0.399 | 13 |
| 16: Bwawa la  Muamachi | Outside ILUMA WMA | Miombo Woodland | 6 | 28 | 4 | 15 | 0.574 | 27 | -0.603 | 26 | 1.325 | 30 |
| 17: Tuliza Moyo | Outside ILUMA WMA | Miombo Woodland | 4 | 30 | 5 | 14 | 0.340 | 30 | -0.581 | 23 | 1.082 | 28 |
| 18: Mavimba Porini | Outside ILUMA WMA | Miombo Woodland | 10 | 27 | 4 | 15 | 0.159 | 32 | -0.568 | 22 | 0.065 | 26 |
| 19: Bwawa la Selessusi | Outside ILUMA WMA | Miombo Woodland | 25 | 25 | 11 | 11 | 0.740 | 25 | -0.749 | 31 | -0.337 | 24 |
| 20: Makingi | Outside ILUMA WMA | Miombo Woodland | 0 | 32 | 2 | 16 | 0.234 | 31 | -0.597 | 25 | 1.185 | 29 |
| 21: Bwawa la Mpunga | Inside ILUMA WMA | Miombo Woodland | 55 | 19 | 12 | 10 | 0.785 | 23 | -0.721 | 30 | -0.344 | 22 |
| 22: Kisaki | Outside ILUMA WMA | Miombo Woodland | 5 | 29 | 5 | 14 | 0.411 | 29 | -0.607 | 27 | 3.805 | 32 |
| 23: Uwanja wa Ndege | Outside ILUMA WMA | Miombo Woodland | 3 | 31 | 6 | 13 | 0.504 | 28 | -0.621 | 29 | 2.781 | 31 |
| 24: Bwawa la Mkwajuni | Inside ILUMA WMA | Miombo Woodland | 45 | 21 | 11 | 11 | 0.841 | 21 | -0.529 | 21 | -0.340 | 23 |
| 25: Bwawa la Mamba  Luhogi | Inside ILUMA WMA | Miombo Woodland | 48 | 20 | 15 | 7 | 0.836 | 22 | -0.850 | 32 | 0.144 | 27 |
| 26: Funga | Inside ILUMA WMA | Groundwater forest | 77 | 14 | 13 | 9 | 0.754 | 24 | -0.401 | 16 | -0.366 | 19 |
| 27: Bwawa la Mlenda | Inside ILUMA WMA | Miombo Woodland | 83 | 10 | 15 | 7 | 0.879 | 20 | -0.162 | 11 | -0.408 | 12 |
| 28: Bwawa la Semka | Inside ILUMA WMA | Miombo Woodland | 80 | 13 | 16 | 6 | 0.928 | 15 | 0.587 | 5 | -0.488 | 5 |
| 29: Bwawa la Simba | Nyerere National Park | Groundwater Forest | 97 | 3 | 11 | 11 | 0.910 | 16 | 2.356 | 3 | -0.549 | 2 |
| 30: Kiboko Zanzibar | Nyerere National Park | Acacia Savanna | 100 | 1 | 16 | 6 | 0.935 | 11 | 2.920 | 1 | -0.519 | 3 |
| 31: Zanzibar | Nyerere National Park | Miombo Woodland | 92 | 4 | 14 | 8 | 0.949 | 7 | 1.606 | 4 | -0.489 | 4 |
| 32: Bwawa la Moto | Nyerere National Park | Miombo Woodland | 99 | 2 | 18 | 4 | 0.948 | 8 | 2.724 | 2 | -0.590 | 1 |

^a^ Based on personal, subjective opinions of the three investigators based on their impressions over the time spent in the field, with each camp being given a consensus score from 0 to 100%, with 100% representing completely pristine conditions with no evidence of human activities and 0% representing highly degraded areas that have been completely converted into fully domesticated habitats. See sections 2.4.4.4 and 3.4.4 for details.

^b^ Defined as *S* the sum of the total number of species recorded in a particular area (each camp). See sections *2.4.4.2* and *3.4.2* for details.

^c^ Calculates the probability of selecting two individuals from different species within a defined area (camp) and calculated as SDI=1- D, where D = $\sum iPi$ ^2^, where the species proportion(the proportion of a particular species divided by the total number of all species observations) (*P_i_*) is calculated as *P_i_* = *x_i_*/*n*, where *x_i_* is the total number of individuals of species *i* recorded and *n* is the total number of individuals from all species recorded. See sections *2.4.4.3* and *3.4.3* for details.

^d^ Scaled results of Principal Component Analysis of all parameters recorded for wild herbivores, rodents, carnivores, primates, and prosimians. See sections *2.4.4.5* and *3.4.5* for details.

^e^ Scaled results of Principal Component Analysis of all parameters recorded for human, livestock, and wild animals. See sections *2.4.4.6* and *3.4.6* for details.

^f^ Locations ranked from the most species rich, biodiverse, or ecologically intact to the least, based on values from each index.
