# Supplementary material for: A Subjective and Intuitive Approach to Rapid, Holistic Assessment of Natural Ecosystem Integrity Across a Community‐Managed Conservation Area in Southern Tanzania
Source: Ecol Evol. 2025 Mar 2;15(3):e70872. doi: 10.1002/ece3.70872 (PMC11872596; doi:10.1002/ece3.70872)
Supplement: Supplementary file 3 — Data S3. Figure S3.1–S3.3: Additional photographs, complementing those presented in Figure 2, to illustrate different ways in which signs of livestock, humans or wild mammal activity, as well as land cover attributes, were observed and classified; https://doi.org/10.5281/zenodo.12795058. [file ECE3-15-e70872-s006.docx]

**Supplementary figure S3.1:** Photographs illustrating unauthorised activities recorded during data collection in the field. Panel **A** shows active charcoal burning. Timber from felled trees is placed in a pile, covered in soil to create a low oxygen environment, and then set on fire. Panel **B** displays an area that is undergoing active deforestation. The trees that have been felled would be used for charcoal production or for the construction of human settlements or occasionally timber to be sold commercially. Panel **C** shows evidence of unauthorised fishing, in which a dam made of soil and mud was constructed across a large stream to block the flow of water and prevent fish from moving past that point. Also exhibited is a bed net, which had been used to catch fish trapped within the dammed pond. Panel **D** exhibits items seized from a meat poacher during a routine patrol. Bicycles are an efficient mode of transport through the WMA for both people and goods. Sufficient supplies for up to a week and equipment used to capture wild animals were in the sack behind the bicycle.

**Supplementary figure S3.2:** Panels **A** and **B** both illustrate an unauthorised human activity taking place in the ILUMA WMA that was not anticipated a priori, which is presented an example of an unusual activity. Here, a form of homemade alcohol known as *gongo* is being produced through distillation with grain cultivated illegally inside the conservation area. As with fish poaching, a river is blocked which causes knock-on issues for the aquatic ecosystem. Pollution of the watercourse is evident here.

**Supplementary figure S3.3:** Images showing how the impression of land cover for rice farming and other tillage agriculture was recorded. Both panels **A** and **B** display rice agriculture in different stages. The rice in image **A** is still growing and is lush green. Image **B** depicts how this same area looked once the rice had been harvested and the dry season began. Panel **C** demonstrates the other forms of tillage farming detected in ILUMA. Here, maize is beginning to grow in deforested and cleared land. The mature trees in the background depict what the area looked prior to agricultural use. Panel **D** illustrates an unauthorised human settlement inside ILUMA structures like this usually accompany agricultural activity such as that showed in image **C**. Note here the level of land clearance in the area surrounding the settlement.
