# Supplementary material for: A Subjective and Intuitive Approach to Rapid, Holistic Assessment of Natural Ecosystem Integrity Across a Community‐Managed Conservation Area in Southern Tanzania
Source: Ecol Evol. 2025 Mar 2;15(3):e70872. doi: 10.1002/ece3.70872 (PMC11872596; doi:10.1002/ece3.70872)
Supplement: Supplementary file 5 — Data S5. Data collection key devised during preliminary study in October and November 2021 and used throughout the course of the field study to inform accurate and consistent recording of data; https://doi.org/10.5281/zenodo.10955638. [file ECE3-15-e70872-s010.docx]

**Table S5.** Data collection key devised during preliminary study in October and November 2021 and used throughout the course of the field study to inform accurate and consistent recording of various humans, livestock and animals, their activities and land cover attributes.

| **Entity or Activity Observed (EAO)** | **Observation Category (OC)** | **Additional Observation Attributes (AOA)** | **Observation Count or Class (OCC)** |
| --- | --- | --- | --- |
| **1X** “As you go” instances of specific human land and/or natural resources usages. | | | |
| **11**: Livestock Herding |  |  | **1**: Active, **2**: Recent, **3**: Old, **9**: Not determined or recorded |
| **12**: Charcoal Burning |  |  | **1**: Active, **2**: Recent, **3**: Old, **9**: Not determined or recorded |
| **13**: Timber Harvesting |  |  | **1**: Active, **2**: Recent, **3**: Old, **9**: Not determined or recorded |
| **14**: Fishing |  |  | **1**: Active, **2**: Recent, **3**: Old, **9**: Not determined or recorded |
| **15**: Hunting |  |  | **1**: Active, **2**: Recent, 3: Old, **9**: Not determined or recorded |
| **16**: Human Settlement |  | **1,2,3…10** structures [n], 11-20 structures **[20],** 21-50 structures **[50],** >50 structures **[100]**, not determined or recorded **[9]** | **1**: Active, **2**: Recent, **3**: Old, **9**: Not determined or recorded |
| **17**: Water Body |  |  | **1**: Stagnant waterbody, **2**: Puddle(s) outside a seasonal streambed, **3:**  Puddle(s) outside a seasonal streambed **4**: Flowing stream, **5**: Flooded valley |
| **18:** Human dug well |  |  | **1**: Active, **2**: Recent, **3**: Old, **9**: Not determined or recorded |
| **19:** Meat Poaching |  |  | **1:** Active**, 2:** Recent, **3:** Old**, 9:** Not determined or recorded |
| **2X**: “As you go” sightings, tracks and signs of humans and animals. | | | |
| **21**: Humans | 1: Direct sighting, 2: Tracks, 3: Spoor, **4**: Other signs (Explain in notes) | **1**: ILUMA personnel, **2**: NNP Rangers, **3**: Hunting company staff, **4**: Other authorized personnel from ILUMA and other authorized institutions, **5**: Community legal presence inside conservation area, **6**: Community legal presence outside the conservation area, **7**: Charcoal transport, **8**: Timber harvesting, **9**: Timber transport, **10**: Fish harvesting, **11**: Fish transport, **12**: Community illegal presence inside the conservation area, **13:** Charcoal burner. | Distance (m) from track for direct observations, or age for tracks and signs: **1**: Active, **2**: Recent, **3**: Old, **9**: Not determined or recorded |
| **22**: Livestock and work animals | **1**: Direct sighting, **2**: Tracks, **3**: Spoor, **4**: Other | **1**: Cattle, **2**: Goats, **3**: Sheep, **4**: Rooster, **5**: Chicken, **6**: Dog | Distance (m) from track for direct observations, or age for tracks and signs: **1**: Active, **2**: Recent, **3:** Old, **9**: Not determined or recorded |
| **23**: Wild Herbivores | **1**: Direct sighting, **2**: Tracks, **3**: Spoor, **4**: Other signs (Explain in notes) | 1X: Reduncini (**11**: Reedbuck, **12**: Waterbuck, **13**: Puku), 2X: Spiral Horned Antelopes (**21**: Bushbuck, **22**: Eland, **23**: Greater Kudu) 3X: Alcephalines (**31**: Hartebeest, **32**: Wildebeest), 4X: Hippotragini (**41**: Sable Antelope), 5X: Suidae (**51**: Warthog, **52**: Bushpig), 6X Plantigrade ungulates (**61**: Hippo, **62**: Elephant), 7X: Bovinae (**71**: African Buffalo), 8X: Equidae (**81**: Plains Zebra), 9X: Neotragini (**91**: Suni), 10X: Cephalophini (**101**: Common Duiker, **102**: Red Duiker), 11X: Aepycerotini (**111**: Common Impala), 12X: Madoquini (**121**: Dikdik), 13X: Raphicerini (**131**: Sharpe's Grysbok) | Distance (m) from track for direct observations, or age for tracks and signs: **1**: Active, **2**: Recent, **3**: Old, **9**: Not determined or recorded |
| **24**: Wild Carnivores | 1: Direct sighting, 2: Tracks, 3: Spoor, 4: Other signs (Explain in notes) | 1X: Pantherinae (**11**: Lion, **12**: Leopard), 2X: Felidae (**21**: African wild cat), 3X: Viverridae (**31**: African Civet, **32**: Cape Genet), 4X: Hyaenidae (**41**: Spotted Hyena), 5X: Canidae (**51**: African wild dog, **52**: Side - Striped Jackal), 6X: Herpestidae (**61**: Slender Mongoose, **62**: Banded Mongoose **63:** Water Mongoose, **64:** White Tailed Mongoose), 7X: Mustelids (**71**: Honey Badger), 8X Lutrinae ( **81**: African Clawless Otter) | Distance (m) from track for direct observations, or age for tracks and signs: **1**: Active, **2**: Recent, **3**: Old, **9**: Not determined or recorded |
| **25**: Wild Primates and Prosimians | **1**: Direct sighting, **2**: Tracks, **3**: Spoor, **4**: Other | **1**: Lesser Galago, **2**: Greater Galago, **3**: Baboon, **4**: Vervet, **5**: Blue Monkey | Distance (m) from track for direct observations, or age for tracks and signs: **1**: Active, **2**: Recent, **3**: Old, **9**: Not determined or recorded |
| **26**: Rodents | **1**: Direct sighting, **2**: Tracks, **3**: Spoor, **4**: Other | 1: Hystricidae (**11**: Porcupine, **12**: Giant Cane Rat) | Distance (m) from track for direct observations, or age for tracks and signs: **1**: Active, **2**: Recent, **3**: Old, **9**: Not determined or recorded |
| **27:** Macroscelidea | **1**: Direct sighting, **2**: Tracks, **3**: Spoor, **4**: Other | 1: Rhynchoninae (**11:** Sengei) | Distance (m) from track for direct observations, or age for tracks and signs: **1**: Active, **2**: Recent, **3**: Old, **9**: Not determined or recorded |
| **28:** Tubulidentata | **1**: Direct sighting, **2**: Tracks, **3**: Spoor, **4**: Other | 1: Orycteropodidae (**11:** Aardvark) | Distance (m) from track for direct observations, or age for tracks and signs: **1**: Active, **2**: Recent, **3**: Old, **9**: Not determined or recorded |
| **3X**: Summary impressions of land cover attributes averaged over entire segment. | | | |
| **31**: Rice farming |  |  | **1**: 0%, **2**: 1 - 25%, **3**: 26 - 50%, **4**: 51 - 75%, **5**: 76 - 100% |
| **32**: Other Tillage Crops |  |  | **1**: 0%, **2**: 1 - 25%, **3**: 26 - 50%, **4**: 51 - 75%, **5**: 76 - 100% |
| **33**: Grass and forb height |  |  | **1:** <1ft, **2:** 1 - 3ft, **3:** 3 -6ft, **4:** 6ft+ |
| **34:** Visibility of obstruction by Shrubs, Bushes and Trees |  |  | **1**: 0%, **2**: 1 - 25%, **3**: 26 - 50%, **4**: 51 - 75%, **5**: 76 - 100% |
| **35:** Ground Type |  |  | **1**: Hard & Dry, **2:** Sand**, 3:** Damp Soil, **4**: Mud, **5:** Water**, 6:** Rock**, 7:** Gravel & Pebbles |
