# Supplementary material for: A Subjective and Intuitive Approach to Rapid, Holistic Assessment of Natural Ecosystem Integrity Across a Community‐Managed Conservation Area in Southern Tanzania
Source: Ecol Evol. 2025 Mar 2;15(3):e70872. doi: 10.1002/ece3.70872 (PMC11872596; doi:10.1002/ece3.70872)
Supplement: Supplementary file 6 — Data S6. Criteria defining terms used in data dictionary key; https://doi.org/10.5281/zenodo.10955643. [file ECE3-15-e70872-s007.docx]

**Table S6.** Criteria defining terms used in data dictionary key.

| **Direct sighting:** Live animal or human actually seen by one or more members of the field team. |
| --- |
| **Tracks:** Prints (animal or human) seen by the data recorder on the ground on the immediate path being followed. |
| **Spoor:** Faecal excrement seen by the data recorder on the on the immediate path being followed. |
| **Other signs:** Any sign of animal activity that undoubtedly links to a specific species (elephant pulling bark from a tree or water mongoose cracking shells for example). |
| **Active:** Activity was directly observed or estimated to have occurred within the last 24 hours. |
| **Recent:** Activity estimated to have occurred between 2 and 5 days ago. |
| **Old:** Activity estimated to have occurred between 6 days and 6 months ago. |
