# Supplementary material for: A Subjective and Intuitive Approach to Rapid, Holistic Assessment of Natural Ecosystem Integrity Across a Community‐Managed Conservation Area in Southern Tanzania
Source: Ecol Evol. 2025 Mar 2;15(3):e70872. doi: 10.1002/ece3.70872 (PMC11872596; doi:10.1002/ece3.70872)
Supplement: Supplementary file 7 — Data S7. Data collection sheet developed during preliminary investigation in October and November 2021 and used for all data collection in both transect segment surveys and camp radial surveys throughout the course of the study https://doi.org/10.5281/zenodo.10955725. [file ECE3-15-e70872-s002.docx]

**Table S7.** Data collection sheet developed during preliminary investigation in October and November 2021 and used for all data collection in both transect segment surveys and camp radial surveys throughout the course of the study.

| **Form Type (FT):** Land Use Observations (LUO) | | | | **Date (DT):** / / | | **GPS Identifier (GID):** | |  | **Form Serial Number (SEN):** | | |  |
| --- | --- | --- | --- | --- | --- | --- | --- | --- | --- | --- | --- | --- |
| **Transect Start Camp Number (TSCNO):** | | | | **Transect Start Camp Name or Description (TSCND):** | | | | |  |  |  |  |
| **Transect Segment Number (TSN):** | | |  |  | **Transect Start GPS Way Point Number (TSGWPN):** | | | | |  |  |  |
| **Transect Segment Page Number (TSPN):** | | | |  | **Transect End GPS Way Point Number (TEGWPN):** | | | | |  |  |  |
| **Form Row (FR)** | **Entity or Activity Observed (EAO)** | **Observation Category (OC)** | **Additional observation attributes (AOA)** | **Observation count or class (OCC)** | **Observation Notes (ONO):** Specify details that add qualitative insight or enable post-hoc classification | | | | | | | |
| 1 |  |  |  |  |  |  |  |  |  |  |  |  |
| 2 |  |  |  |  |  |  |  |  |  |  |  |  |
| 3 |  |  |  |  |  |  |  |  |  |  |  |  |
| 4 |  |  |  |  |  |  |  |  |  |  |  |  |
| 5 |  |  |  |  |  |  |  |  |  |  |  |  |
| 6 |  |  |  |  |  |  |  |  |  |  |  |  |
| 7 |  |  |  |  |  |  |  |  |  |  |  |  |
| 8 |  |  |  |  |  |  |  |  |  |  |  |  |
| 9 |  |  |  |  |  |  |  |  |  |  |  |  |
| 10 |  |  |  |  |  |  |  |  |  |  |  |  |
| 11 |  |  |  |  |  |  |  |  |  |  |  |  |
| 12 |  |  |  |  |  |  |  |  |  |  |  |  |
| 13 |  |  |  |  |  |  |  |  |  |  |  |  |
| 14 |  |  |  |  |  |  |  |  |  |  |  |  |
| 15 |  |  |  |  |  |  |  |  |  |  |  |  |
| 16 |  |  |  |  |  |  |  |  |  |  |  |  |
| 17 |  |  |  |  |  |  |  |  |  |  |  |  |
| 18 |  |  |  |  |  |  |  |  |  |  |  |  |
| 19 |  |  |  |  |  |  |  |  |  |  |  |  |
| 20 |  |  |  |  |  |  |  |  |  |  |  |  |
| 21 |  |  |  |  |  |  |  |  |  |  |  |  |
| 22 |  |  |  |  |  |  |  |  |  |  |  |  |
| 23 |  |  |  |  |  |  |  |  |  |  |  |  |
