# Supplementary material for: A Subjective and Intuitive Approach to Rapid, Holistic Assessment of Natural Ecosystem Integrity Across a Community‐Managed Conservation Area in Southern Tanzania
Source: Ecol Evol. 2025 Mar 2;15(3):e70872. doi: 10.1002/ece3.70872 (PMC11872596; doi:10.1002/ece3.70872)
Supplement: Supplementary file 9 — Data S9. Figure S9.1–S9.4: Maps of the ILUMA WMA, respectively displaying the Species Richness Index (SRI), Simpson’s Index of Diversity (SID), Objective Wild Animal Community Integrity Index (OWACII) and Objective Natural Ecosystem Integrity Index (ONEII) scores estimated for each camp as described in sections 2.4 and 2.5; https://doi.org/10.5281/zenodo.10955759. [file ECE3-15-e70872-s009.docx]

**Supplementary Figure S9.1:** Map of the ILUMA WMA, displaying the Species Richness Index (SRI) scores estimated for each camp as described in sections 2.4 and 2.5.

**Supplementary Figure S9.2:** Map of the ILUMA WMA, displaying the Simpson’s Index of Diversity (SID) scores estimated for each camp as described in sections 2.4 and 2.5.

**Supplementary Figure S9.3:** Map of the ILUMA WMA, displaying the Objective Wild Animal Community Integrity Index (ONACII) scores estimated for each camp as described in sections 2.4 and 2.5.

**Supplementary Figure S9.4:** Map of the ILUMA WMA, displaying the Objective Natural Ecosystem Integrity Index (ONEII) scores estimated each camp as described in sections 2.4 and 2.5.
