# Supplementary material for: A Subjective and Intuitive Approach to Rapid, Holistic Assessment of Natural Ecosystem Integrity Across a Community‐Managed Conservation Area in Southern Tanzania
Source: Ecol Evol. 2025 Mar 2;15(3):e70872. doi: 10.1002/ece3.70872 (PMC11872596; doi:10.1002/ece3.70872)
Supplement: Supplementary file 10 — Data S10. Table S10.1–S10.3; Results of univariate and multivariate generalized linear modeling analyses of scaled human activity indicators as respective determinants of the estimated Species Richness Index (SRI), Simpson’s Index of Diversity (SID) and Objective Wild Animal Community Integrity Index (OWACII) for each camp surveyed; https://doi.org/10.5281/zenodo.10955769. [file ECE3-15-e70872-s008.docx]

**Supplementary Table S10.1:** Results of univariate and multivariate generalized linear modelling analyses of scaled human activity indicators as determinants of the estimated Species Richness Index (SRI) for each camp surveyed, with statistically significant associations highlighted in bold. The best fit models identified for this SRI outcome all assumed a Gaussian distribution and an inverse link function for the dependent variable, so positive values therefore indicate a negative association between the independent and dependent variable.

| Activity | Univariate | | | Multivariate | | |
| --- | --- | --- | --- | --- | --- | --- |
|  |  | | |  | | |
|  | β **±** SEM | t | P | β **±** SEM | t | P |
|  |  |  |  |  |  |  |
| Livestock Herding | **0.0186 ± 0.0056** | **3.348** | **0.0022** | **0.0186 ± 0.0056** | **3.348** | **0.0022^a^** |
| Charcoal Burning | **0.0273 ± 0.0115** | **2.381** | **0.0238** | 0.0038 **±** 0.0047 | 0.806 | 0.4271^b^ |
| Timber Harvesting | **0.0049 ± 0.0018** | **2.636** | **0.0132** | 0.0016 **±** 0.0010 | 1.508 | 0.1425^b^ |
| Fishing | 0.0000 **±** 0.0034 | -0.008 | 0.9940 | 0.0046 **±** 0.0028 | 1.649 | 0.1099^b^ |
| Hunting | **0.0030 ± 0.0010** | **2.923** | **0.0065** | 0.0010 **±** 0.0007 | 1.504 | 0.1434^b^ |
| Human Settlement | 0.0150 **±** 0.0080 | 1.871 | 0.0712 | 0.0017 **±** 0.0060 | 0.278 | 0.7830^b^ |
| Well | **0.0376 ± 0.0143** | **2.634** | **0.0132** | -0.0026 **±** 0.0065 | -0.404 | 0.6890^b^ |
| Meat Poaching | 0.0009 **±** 0.0026 | 0.336 | 0.7390 | -0.0015 **±** 0.0018 | -0.825 | 0.4160^b^ |
| Human Presence | 0.0667 **±** 0.0508 | 1.314 | 0.1989 | 0.0657 **±** 0.0633 | 1.037 | 0.3083^b^ |
| Rice Farming | **0.0117 ± 0.0040** | **2.940** | **0.0063** | 0.0031 **±** 0.0030 | 1.031 | 0.3111^b^ |
| Other Tillage Farming | **0.0132 ± 0.0051** | **2.587** | **0.0148** | 0.0033 **±** 0.0043 | 0.759 | 0.4543^b^ |

SEM: Standard error of the mean for the β coefficient estimate.

^a^ As estimated from the final, most parsimonious best-fit GLM, in which this was included on the basis of either being a significant (P≤0.05) determinant of SRI or approached significance (P≤0.10) as a determinant of SRI and significantly (P≤0.05) improved the goodness of fit based on the estimated Akaike Information Criterion (AIC).

^b^ As estimated from the last GLM in which this variable was still included before being removed on the basis that it was not a significant determinant of the SRI (P>0.05) or approached significance (P≤0.10) but did not significantly improve the goodness of fit based on the estimated AIC (P>0.05).

**Supplementary Table S10.2:** Results of univariate and multivariate generalized linear modelling analyses of scaled human activity indicators as determinants of the estimated Simpson’s Index of Diversity (SID) for each camp surveyed, with statistically significant associations highlighted in bold. The best fit models identified for this SID outcome all assumed a Gamma distribution and log link function for the dependent variable.

| Activity | Univariate | | | Multivariate | | |
| --- | --- | --- | --- | --- | --- | --- |
|  |  | | |  | | |
|  | β **±** SEM | T | P | β **±** SEM | t | P |
|  |  |  |  |  |  |  |
| Livestock Herding | **-0.253 ± 0.042** | **-6.01** | **<0.0001** | **-0.218 ± 0.051** | **-4.254** | **0.0002^a^** |
| Charcoal Burning | -0.113 **±** 0.062 | -1.842 | 0.0754 | 0.031 **±** 0.036 | 0.842 | 0.4069^b^ |
| Timber Harvesting | **-0.089 ± 0.043** | **-2.054** | **0.0488** | -0.014 **±** 0.025 | -0.540 | 0.5938^c^ |
| Fishing | -0.033 **±** 0.047 | -0.697 | 0.4913 | **-0.082 ± 0.030** | **-2.788** | **0.0094^a^** |
| Hunting | -0.029 **±** 0.045 | -0.659 | 0.5151 | 0.004 **±** 0.025 | 0.180 | 0.8586^b^ |
| Human Settlement | **-0.191 ± 0.058** | **-3.314** | **0.0024** | -0.046 **±** 0.052 | -0.883 | 0.3850^b^ |
| Well | **-0.245 ± 0.074** | **-3.306** | **0.0025** | 0.052 **±** 0.070 | 0.742 | 0.4642^b^ |
| Meat Poaching | -0.039 **±** 0.044 | -0.893 | 0.379 | 0.001 **±** 0.025 | 0.047 | 0.9632^b^ |
| Human Presence | -0.365 **±** 0.089 | -4.109 | **0.0003** | -0.129 **±** 0.074 | -1.739 | 0.0931^a^ |
| Rice Farming | **-0.179 ± 0.044** | **-4.100** | **0.0003** | 0.021 **±** 0.041 | 0.498 | 0.6225^b^ |
| Other Tillage Farming | **-0.218 ± 0.043** | **-5.078** | **<0.0001** | -0.020 **±** 0.051 | -0.396 | 0.6950^b^ |

SEM: Standard error of the mean for the β coefficient estimate.

^a^ As estimated from the final, most parsimonious best-fit GLM, in which this was included on the basis of either being a significant (P≤0.05) determinant of SID or approached significance (P≤0.10) as a determinant of SID and significantly (P≤0.05) improved the goodness of fit based on the estimated Akaike Information Criterion (AIC).

^b^ As estimated from the last GLM in which this variable was still included before being removed on the basis that it was not a significant determinant of the SID (P>0.05) or approached significance (P≤0.10) but did not significantly improve the goodness of fit based on the estimated AIC (P>0.05).

**Supplementary Table S10.3:** Results of univariate and multivariate generalized linear modelling analyses of scaled human activity indicators as determinants of the estimated Objective Wild Animal Community Integrity Index (OWACII) for each camp surveyed, with statistically significant associations highlighted in bold. The best fit models identified for this OWACII outcome all assumed a Gaussian distribution and identity link function for the dependent variable.

| Activity | Univariate | | | Multivariate | | |
| --- | --- | --- | --- | --- | --- | --- |
|  |  | | |  | | |
|  | β **±** SEM | t | P | β **±** SEM | t | P |
|  |  |  |  |  |  |  |
| Livestock Herding | **-0.513 ± 0.157** | **-3.275** | **0.0027** | **-0.442 ± 0.151** | **-2.918** | **0.0067^a^** |
| Charcoal Burning | -0.378 **±** 0.169 | -2.233 | 0.1691 | -0.129 **±** 0.172 | -0.751 | 0.4591^b^ |
| Timber Harvesting | 0.036 **±** 0.183 | 0.199 | 0.844 | 0.191 **±** 0.154 | 1.239 | 0.2255^b^ |
| Fishing | 0.266 **±** 0.176 | 1.51 | 0.141 | 0.136 **±** 0.153 | 0.888 | 0.3822^b^ |
| Hunting | -0.062 **±** 0.182 | -0.339 | 0.737 | -0.437 **±** 0.153 | -0.029 | 0.9775^b^ |
| Human Settlement | **-0.374 ± 0.169** | **-2.21** | **0.0349** | -0.131 **±** 0.172 | -0.761 | 0.4532^b^ |
| Well | -0.346 **±** 0.171 | -2.022 | 0.0522 | 0.104 **±** 0.231 | 0.450 | 0.6561^b^ |
| Meat Poaching | **-0.425 ± 0.165** | **-2.568** | **0.0154** | **-0.329 ± 0.151** | **-2.170** | **0.0384^a^** |
| Human Presence | -0.256 **±** 0.177 | -1.453 | 0.157 | 0.248 **±** 0.222 | 1.121 | 0.2718^b^ |
| Rice Farming | **-0.507 ± 0.157** | **-3.218** | **0.0031** | -0.255 **±** 0.206 | -1.235 | 0.2272^b^ |
| Other Tillage Farming | **-0.465 ± 0.162** | **-2.877** | **0.0073** | -0.171 **±** 0.222 | -0.767 | 0.4494^b^ |

SEM: Standard error of the mean for the β coefficient estimate.

^a^ As estimated from the final, most parsimonious best-fit GLM, in which this was included on the basis of either being a significant (P≤0.05) determinant of OWACII or approached significance (P≤0.10) as a determinant of OWACII and significantly (P≤0.05) improved the goodness of fit based on the estimated Akaike Information Criterion (AIC).

^b^ As estimated from the last GLM in which this variable was still included before being removed on the basis that it was not a significant determinant of the OWACII (P>0.05) or approached significance (P≤0.10) but did not significantly improve the goodness of fit based on the estimated AIC (P>0.05).
